# Of von Willebrand factor and platelets

**DOI:** 10.1007/s00018-014-1743-8

**Published:** 2014-10-09

**Authors:** Marijke Bryckaert, Jean-Philippe Rosa, Cécile V. Denis, Peter J. Lenting

**Affiliations:** 1 INSERM U770, Hôpital Bicêtre, 80 rue du Général Leclerc, 94276 Le Kremlin Bicêtre Cedex, France; 2 Université Paris-Sud, Le Kremlin Bicêtre, France

**Keywords:** Platelets, von Willebrand factor, Glycoprotein Ib-IX-V, Hemostasis, Thrombosis

## Abstract

Hemostasis and pathological thrombus formation are dynamic processes that require multiple adhesive receptor-ligand interactions, with blood platelets at the heart of such events. Many studies have contributed to shed light on the importance of von Willebrand factor (VWF) interaction with its platelet receptors, glycoprotein (GP) Ib-IX-V and αIIbβ3 integrin, in promoting primary platelet adhesion and aggregation following vessel injury. This review will recapitulate our current knowledge on the subject from the rheological aspect to the spatio-temporal development of thrombus formation. We will also discuss the signaling events generated by VWF/GPIb-IX-V interaction, leading to platelet activation. Additionally, we will review the growing body of evidence gathered from the recent development of pathological mouse models suggesting that VWF binding to GPIb-IX-V is a promising target in arterial and venous pathological thrombosis. Finally, the pathological aspects of VWF and its impact on platelets will be addressed.

## Introduction

Platelets produced by the cytoplasmic fragmentation of megakaryocytes (MKs) are required for human survival due to their ability to arrest bleeding. After adhering to vascular lesions, platelets rapidly recruit additional platelets, till blood stops flowing, achieving hemostasis. Dysregulation of this system has devastating consequences. Excessive accumulation of platelets at sites of atherosclerotic plaque rupture is one of the key pathogenic events triggering arterial thrombus formation, leading to acute myocardial infarction or ischemic stroke. On the other hand, unstoppable hemorrhage might occur if the hemostatic system fails to react appropriately upon injury. In the last decades, the challenge has been and still is, to identify platelet receptors, signaling pathways and cytoskeleton reorganization events involved in platelet adhesion, activation, aggregation and pro-coagulant activity to identify potential targets for antiplatelet drugs. Traditional research using biochemical and molecular biology approaches has paved the way and helped identifying basic mechanisms involved in platelet adhesion, activation and aggregation. However, considerable progress has been made in the last 20 years with the availability of new imaging techniques and the generation of genetically-modified animal models. These advances have led to a better understanding of the spatial and temporal relationships between specific platelet adhesion and activating events and the influence of the rapidly changing shear environment during thrombus development. Indeed, in vivo, the dynamics of thrombus formation and development was open to question. In the classical model of platelet engagement, mostly derived from in vitro studies, platelet adhesion on the reactive subendothelial matrix proteins (von Willebrand factor (VWF), collagens type I, III and VI) appears to be the initiating event for arterial thrombus formation, which occurs via the specific platelet receptors glycoproteins (GP) Ib-IX-V or GPVI, dependent on rheological conditions [1]. Under high shear, it was clearly established that the VWF/GPIb-IX-V interaction is the predominant receptor-ligand interaction initiating platelet adhesion. For stable adhesion, the collagen receptors (GPVI and α2β1 integrin) as well as the fibronectin receptor (α5β1 integrin) are required. In this model, thrombus formation involves the release of soluble platelet agonists such as ADP and thromboxane A_2_ (TXA_2_) and the activation of the αIIbβ3 integrin, the receptor for fibrinogen and VWF, allowing platelet–platelet interactions. Finally, stabilization of the thrombus is dependent on thrombin generation and fibrin polymerization. A revised model of platelet engagement, emerging from in vivo studies has revealed subtle differences such as the heterogeneity of thrombus formation. Indeed, a stable “core” of the thrombus is composed of fully activated platelets undergoing marked morphological changes, whereas the outer shell of the thrombus is sensitive to rheological conditions and consists of aggregates of discoid platelets which are “weakly” activated. The challenge of the last years has been to target processes associated with the different steps of thrombus formation, propagation and stabilization offering thrombotic protection with a minor bleeding risk. The interaction of VWF with GPIb-IX-V is placed high on the list of platelet targets of clinical interest. In this review, we will summarize the recent advances on the molecular mechanisms involved in the interaction of VWF with GPIb-IX-V and its role on arteriolar but also in venous thrombosis.

## Von Willebrand factor: gene, structure, biosynthesis, secretion and clearance

### The VWF gene and protein

The gene encoding VWF is located on the short-arm of chromosome 12, encompassing 52 exons dispersed over 179 kb [2]. A non-functional pseudogene corresponding to exons 23–34 (97 % homology) has been identified on chromosome 22. Transcription of the VWF gene results in an 8.9 kb mRNA that is subsequently translated into a pre-pro-VWF precursor protein of 2,813 amino acids. This precursor is composed of a signal peptide of 22 amino acids (aa 1–22; exons 2–3. Indeed, exon 1 is not translated into a protein), a propeptide of 741 amino acids (previously referred to as VWF-antigen II; aa 23–763; exons 3–18) and a mature subunit of 2,050 amino acids (aa 764–2,813; exons 19–52) [3].

**Fig. 1:**
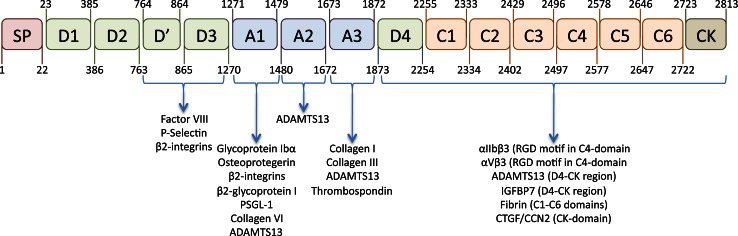
Schematic representation of the VWF domain architecture and location of interactive sites. The molecular architecture of VWF is characterized by a distinct domain structure. The domain organization recently proposed by Zhou et al. [15] is presented in this figure. In addition, the location of the interactive sites for various VWF-binding proteins is indicated [163]. IGFBP7: Insulin growth factor-binding protein-7; CTGF/CCN2: Connective tissue growth factor

The architecture of the VWF protein distinguishes 4 different domain structures that are arranged in the following order: D1-D2-D’-D3-A1-A2-A3-D4-C1-C2-C3-C4-C5-C6-CK (Fig. 1) [4]. Within this structure, the D1-D2 domains represent the propeptide, while the D’-CK portion represents the mature VWF subunit.

### Post-translational processing of VWF

During and following its translation, a series of events occur that are pertinent to the structure of the VWF protein. First, in the endoplasmic reticulum, two pre-VWF subunits engage into a dimeric structure via disulfide bridge formation between C-terminal CK-domains [5]. Subsequently, VWF assembles into a multimeric structure via additional disulfide bridging between N-terminal D’-D3 domains. This latter process requires the presence of the propeptide, which not only aligns the D’-D3 regions (“zipper-model”) but also catalyzes the formation of the disulfide bonds via its protein disulfide isomerase-activity [6]. Of note, the propeptide is separated from the mature subunit via proteolytic processing by furin in the trans-Golgi network [7]. Taken together, multimerization of VWF leads to the production of differentially sized VWF multimers, varying from dimers to multimers that contain as many as 60 subunits.

A second important event in the maturing of the VWF protein is its glycosylation. In total, a pro-VWF subunit contains 17 *N*-linked glycans, four within the propeptide and 13 within the mature subunit [8]. In addition, VWF contains 10 *O*-linked glycans, all of which being located in the mature VWF subunit [9]. Both *O*- and *N*-linked glycans are characterized by a remarkable diversity and >300 N-linked glycan structures have been determined. The majority of these glycans is sialylated. Importantly, both *N*- and *O*-linked glycans present on the mature VWF subunit (but not those on the propeptide) can carry ABO(H) blood group carbohydrate determinants [10]. On average, 13 % of the *N*-linked (1–2 *per* subunit) and 1 % of the *O*-linked (1 *per* 10 subunits) harbor these blood group determinants [8, 9].

### Storage and secretion of VWF

The production of VWF is restricted to MKs and endothelial cells [11, 12]. In both cell types, VWF is targeted to storage organelles: α-granules in MKs/platelets and Weibel-Palade bodies (WPBs) in endothelial cells. Whereas the formation of WPBs is strictly dependent on the presence of VWF, this is not the case for α-granules, which may develop in the absence of VWF [13]. The formation of WPBs is a complex process that requires a specific folding of the VWF protein. VWF multimers associated with its propeptide assemble into a helicoidal structure, a step that allows a 100-fold reduction of its spacial volume [14]. The interior of the right-handed helical tubules comprises the propeptide/D’D3 domains while the remainder of the protein (A1-CK region) sticks out. Advanced electron-microscopy imaging has recently been used to reveal that the A1-CK region folds into a bouquet-like structure, with the different domains being aligned in a side-by-side manner [15]. Probably, the A1-CK-spikes determine the regular spacing between the tubules that characterize the electron-microscopic images of WPBs [16]. In addition, the spacing between the tubules also allows the incorporation of other WPB-residents, proteins such as P-selectin, interleukin-8, angiopoietin-2, osteoprotegerin and many more [17]. The presence of co-residential proteins in WPBs is shared with α-granules, which also consist of multiple proteins. Moreover, WPBs and α-granules have in common that not all of the storage organelles have a similar content [18, 19].

Release of VWF from α-granules requires activation of the platelets, and no constitutive release from platelets has been observed. In contrast, VWF is released from endothelial cells in both a constitutive and regulated fashion [20]. Constitutive release is now believed to originate from the fusion of single WPBs with the endothelial cell membrane. Indeed, WPBs move around within the endothelial cells in non-directed trajectories, and such movements drive single WPBs to the cellular periphery, allowing them to release their contents into the extracellular space [21]. This process is the predominant source of circulating VWF, but is insufficient to generate the long endothelial cell-anchored VWF bundles that serve as an adhesive surface for platelets. Such VWF strings only form upon endothelial stimulation provoking a massive release of WPBs [22]. Agonist-induced stimulation of endothelial cells triggers the formation of VWF-enriched patches, signifying the fusion of multiple WPBs into secretory pods [23]. Upon fusion of these pods with the cellular membrane, bundles of assembled VWF multimers are released into the circulation. This multistep event depends on the action of many proteins associated with vesicle secretion, including members of the Rab- and SNARE-protein families [20].

Apart from the classical secretory pathway, an alternative pathway for the secretion of WPBs has recently been identified. Torisu and colleagues observed that WPBs are often in close proximity or even inside autophagosomes [24]. Further analysis revealed that inhibition of autophagy resulted in reduced VWF release, both in vitro and in vivo. These data point to a regulatory role of autophagosomes in the release of WPBs and additional studies are needed to understand this process in more detail.

### VWF clearance

The average half-life of therapeutical VWF concentrates prepared from large plasma-pools is approximately 16 h, and is relatively conserved between patients [25]. In contrast, the half-life of endogenous VWF that is released following desmopressin-treatment is highly individual-dependent and may vary between 6 and 26 h [26, 27]. The main reason for this apparent discrepancy relates to the individual glycosylation patterns, with particular reference to blood group ABO(H) structures. Individuals with blood group non-O have a longer VWF half-life after desmopressin-treatment than those with blood group O [28]. This longer half-life may also explain the approximately 25 % higher VWF levels in individuals with blood group non-O [29].

The mechanism by which VWF is removed from the circulation has attracted increased attention during the previous decade, not only because of its potential association with the pathogenesis of von Willebrand disease, but also because VWF is a major determinant of the half-life of coagulation factor VIII [30]. Via in vitro and in vivo studies, we now know that VWF is principally eliminated from the circulation by macrophages in liver and spleen, without fully excluding a contribution by other cell types. Involvement of macrophages is perhaps best illustrated by the increased half-life and increased levels of endogenous VWF upon chemical depletion of macrophages [31].

The molecular basis of macrophage-mediated clearance of VWF is only partially uncovered. One receptor that has potentially been linked to VWF clearance is the asialoglycoprotein receptor or Ashwell-receptor. Given that >90 % of the glycan structures are sialylated, it seems conceivable that the Ashwell-receptor plays but a minor role in the basal clearance of VWF. Rather, it participates in the clearance of hypo-sialylated VWF, which may for instance occur upon pathogen infection [32]. Two other receptors that have been linked to VWF clearance are the sialic acid-binding receptor Siglec-5 and the mannose-binding lectin CLEC4 M. Cellular expression of both receptors allows the endocytosis of VWF and their over-expression in murine liver results in reduced VWF levels in these mice [33, 34]. However, no direct clearance experiments have been performed that include these receptors, and therefore their relevance with regard to VWF clearance remains uncertain. Finally, the scavenger-receptor LRP1 has recently been identified as a receptor for VWF [35]. This was unexpected, since LRP1 was previously identified as a receptor for factor VIII, while VWF did not bind to LRP1 and actually inhibited factor VIII-LRP1 interactions [36]. However, mice with a conditioned LRP1-deficiency not only have increased factor VIII levels but also increased VWF levels [35]. In addition, polymorphisms in the LRP1 gene are associated with VWF plasma levels [37]. The answer to these apparently contradictory findings came from experiments revealing that VWF needs to unfold in response to shear stress to interact with LRP1 [35, 38]. Taken together, several receptors have the potential to bind and endocytose VWF. However, so far only LRP1 has been identified to play a role in the basal clearance of VWF. It is likely that in the near future other potential receptors contributing to this process will be identified.

## VWF and platelet receptors

At sites of vascular injury, platelets are recruited to exposed subendothelial extracellular matrix components via specific platelet receptors involved in adhesion and aggregation. In arterioles, VWF is essential for the capture of platelets via two receptors: GPIb-IX-V and αIIbβ3 integrin and requires flowing blood. Depending on shear rates, GPIb-IX-V and αIIbβ3 are also required for thrombus formation. The next parts will be focused on the receptors and the signaling pathways induced by the binding of platelets to VWF.

### GPIb-IX-V

**Fig. 2:**
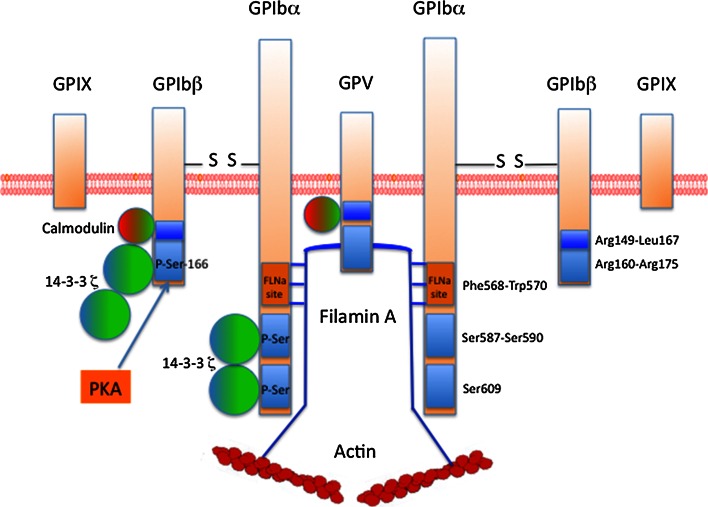
Organization of the GPIb-IX-V complex. GPIb-IX-V is composed of 4 distinct proteins: two trimers consisting each of GPIbα, disulfide-bonded GPIβ, and GPIX, connected to one chain of GPV. The intracellular tail domains of GPIb-IX-V bind different proteins such as Filamin A, calmodulin and the adaptor protein 14-3-3ζ. 14-3-3ζ binds to GPIbα, GPIbβ and GPV, through phospho-serines on GPIbα and GPIbβ (*blue rectangle*). Ser^166^ of GPIbβ is phosphorylated by PKA. FLNa binds to Phe568-Trp570 of GPIbα (*orange rectangle*) and is required for platelet adhesion at high shear rates

GPIb-IX-V is exclusively expressed on the platelet membrane. There are approximately, 25,000 copies of this receptor per platelet. GPIb-IX-V is composed of four distinct transmembrane proteins. The receptor is composed of two chains of GPIbα (135 kDa), 2 GPIbβ (26 kDa), 2 GPIX (20 kDa) and 1 GPV (82 kDa) (2: 2: 2: 1) (Fig. 2). These proteins are encoded by four different genes located to chromosomes 17q12(*GPIBA*), 22q11.2(*GPIBB*), 3q29(*GP5*) and 3q21(*GP9*), respectively. These proteins belong to the leucine-rich repeat (LRR) family. Each subunit is a type I transmembrane protein composed of a large N-terminal extracellular domain containing LRR domains, a transmembrane (TM) helix and a short cytoplasmic tail. The extracellular region of GPIbα is composed of 8 LRR domains, three disulfide bonds, an O- and an N-glycosylated regions and a negative region containing three sulfated tyrosines. VWF binds to the extracellular domain of GPIbα, within amino-acids 1–282, a region that contains seven LRR domains and the disulfide-looped capping sequences of GPIbα. On VWF, the binding site for GPIbα is located in the A1 domain where it becomes exposed under arterial shear, allowing an interaction that is enhanced by increasing shear rates. In static conditions, no binding between VWF and GPIbα is observed. Besides VWF as a main ligand, GPIbα also binds multiple ligands such as thrombospondin, Factor XII, Factor XI, thrombin, High Molecular Weight kininogen, P-selectin and Mac-1. Through its cytoplasmic tail domain (Phe568-Trp570), GPIbα also interacts with the actin-binding protein filamin A (FLNa), phosphoinositide (PI) 3-kinase and the adapter 14-3-3ζ. The phosphorylation of Ser609 of GPIbα is required for the interaction with 14-3-3ζ. GPIbα is non-covalently associated with GPIX and GPV and is connected via disulfide bonds with GPIbβ involving Cys484 and Cys485 in GPIbα and Cys122 in GPIbβ. These disulfide bonds are however not required for proper assembly of the complex. The final structure is thought to consist of two (GPIbα-GPIbβ-GPIX) trimers connected by one GPV. GPIbβ is composed of 181 amino acids and contains 1 LRR domain only. The cytoplasmic domain, which contains 30 amino acids, can associate with calmodulin and 14-3-3ζ. The phosphorylation of Ser166 of GPIbβ by protein kinase A (PKA) is involved in the association with 14-3-3ζ. GPIbβ is essential for the expression of the complex to the membrane. GPIX is composed of 160 amino acids and contains a single LRR sequence in the extracellular domain. The GPIX cytoplasmic tail is short with only eight residues, among which a myristoylated residue (Cys154) and does not seem to associate with any protein. GPV contains 544 amino acids and 15 LRR sequences in its extracellular domain. The intracellular domain of GPV (16 amino acids) binds calmodulin and 14-3-3ζ. Interestingly, GPV seems to be a negative regulator of platelet activation. Indeed, an increase of platelet aggregation induced by thrombin was observed using platelets lacking GPV [39], although another report failed to detect a similar effect [40]. GPV has also been reported as a collagen type I receptor [41].

GPIbα is shed by metalloproteases such as ADAM17, a process that releases a soluble GPIbα fragment termed glycocalicin. ADAM17 cleaves within the GPIbα-based peptide (LRGV^465^LK) through a mechanism that is only partially understood [42]. GPIbα shedding has been shown to be constitutive but it can be increased by activation of protein kinases C (PKC) or inhibition of calmodulin [42, 43]. Shedding leads to decreased receptor density, potentially impacting hemostasis or thrombosis: consequently, the control of shedding could be an alternative to limit platelet reactivity under prothrombotic conditions. Moreover, the ectodomain fragment of GPIbα which is present in normal plasma, could be a potential marker of thrombosis.

The critical importance of the interaction of VWF with GPIb-IX-V for hemostasis was shown in patients with Bernard Soulier syndrome (BSS) who lacked GPIb-IX-V [44] or with patients with von Willebrand disease (VWD) who lacked VWF [45]. In BSS (reviewed in [46] ), the subunits of GPIb-IX-V are usually present, albeit in lower quantities and with some rare exceptions even exhibiting very low levels. *GPIBA* gene is the most frequently affected in BSS but defects in *GPIBB* and *GP9* genes also give rise to BSS. BSS is an autosomal recessive inherited disease and is characterized by macrothrombocytopenia, a defect in ristocetin-induced platelet agglutination and a decrease in platelet adhesion to the subendothelium and in thrombin-induced platelet aggregation. An exception in BSS is the Bolzano variant, due to the A125 V mutation, where platelet GPIb-IX-V is expressed at normal levels but does not bind VWF [47]. The presence of giant platelets and of abnormal membrane complexes in BSS seems to be linked to the absence of the GPIbα cytoplasmic tail and the resulting lack of association with FLNa. Indeed, in a murine model where the extracellular sequence of GPIbα has been replaced by an isolated extracellular domain of the α subunit of the human interleukin-4 receptor, while keeping the GPIbα cytoplasmic sequence intact, a partial rescue of the platelet count and platelet size was observed [48]. Remarkably, giant platelets and thrombocytopenia can also be observed in patients lacking FLNa or with a defect in FLNa content as well as in patients with VWD-type 2B, characterized by an increased affinity of VWF for GPIb-IX-V (see section VWF and pathologies) [49–51]. Altogether, these important data strongly suggest that the VWF/GPIbα/FLNa axis is essential for megakaryopoiesis and proplatelet formation. Further studies are required to investigate the respective role of VWF, GPIbα and FLNa and the mechanisms involved in megakaryopoiesis. This VWF/GPIbα/FLNa axis also plays an essential role in maintaining platelet shape by linking the platelet surface to a sub-membranous network of actin filaments, the platelet membrane skeleton. In the future, patients with FLNa mutations (filaminopathy A) will be promising “models” to study the VWF/GPIbα/FLNa axis in hemostasis and thrombosis and to explore new actors involved in the signaling pathways induced by VWF/GPIbα interaction.

### αIIbβ3 integrin

αIIbβ3 integrin is the second major platelet receptor for VWF. αIIbβ3 is the most abundant surface-expressed integrin (40,000–80,000 copies per platelet) with another pool located in internal membranes and which can be exposed after platelet activation. It is also the most abundant receptor in platelets. αIIb and β3 subunits are 148 and 95 kDa proteins, respectively. Binding of VWF to αIIbβ3 involves the RGD sequence in the carboxyl-terminal region of VWF (residues 2,507–2,509) and requires prior activation of the integrin. Besides VWF, αIIbβ3 integrin binds several other ligands containing an RGD-like sequence: fibrinogen, fibrin, fibronectin and thrombospondin. The important role of αIIbβ3 integrin was demonstrated by the study of patients suffering from the genetic disease Glanzmann thrombasthenia in which platelets lack this integrin and by the study of mice lacking αIIbβ3 [52, 53]. The engagement of αIIbβ3 integrin with fibrinogen and VWF occurs after initial platelet adhesion mediated by VWF/GPIb interaction and is involved in platelet firm adhesion to the subendothelium and in thrombus formation, all these events being dependent on shear.

## VWF/GPIb-IX-V interaction and platelet signaling

VWF binding to GPIb-IX-V induces platelet activation, converting the major integrin αIIbβ3 from a low affinity to a high affinity receptor capable of engaging the C4 domain of VWF. This last step is essential for stable adhesion and for subsequent cytoskeletal reorganization leading to platelet spreading on VWF. The VWF/GPIb-IX-V interaction is also regulated by proteins such as 14-3-3ζ and FLNa which, as already mentioned, are directly associated with GPIb-IX-V (Fig. 2). How VWF/GPIb-IX interaction contributes to platelet activation is still controversial. The difficulties encountered with characterizing the signaling induced by VWF/GPIb-IX interaction arise from the observation that different signaling pathways are activated depending on the levels of shear, either physiological or pathological. Moreover, the variety of models that have been used (1) agglutination/aggregation in the presence of ristocetin or botrocetin or (2) Chinese hamster ovary cells (CHO) transfected with GPIb-IX and integrin αIIbβ3 or (3) adhesion in blood flow at different shears under VWF further, added to confusion.

### Regulation of VWF-GPIb-IX-V interaction

14-3-3ζ is a homodimeric protein which binds to phospho-Ser of GPIbα and GPIbβ. Three binding sites for 14-3-3ζ have been identified: the first is defined by phosphorylated Ser^609^ at the C-terminus of GPIbα, the second by Ser^587^ and Ser^590^ in the cytoplasmic tail of GPIbα [54] and the third by Ser^166^ of GPIbβ phosphorylated by PKA [55]. Dephosphorylation of GPIbβ (Ser^166^) induces activation of VWF-GPIb-IX interaction whereas dephosphorylation of GPIbα (Ser^609^) or blockade of 14-3-3ζ/GPIbα interaction inhibits VWF binding and VWF-mediated platelet adhesion under flow conditions [56]. Du et al. [57] suggested that dephosphorylation of GPIbβ induces dissociation of 14-3-3ζ from GPIbβ therefore allowing 14-3-3ζ dimer to interact with two sites in GPIbα to form the “active state” of GPIb-IX/14-3-3ζ. Altogether these results suggest that 14-3-3ζ/GPIbα interaction could be a potential target for antithrombotic drug development.

FLNa, another protein associated with GPIbα, is also essential for platelet functions induced by VWF. FLNa can be phosphorylated at Ser^2152^ by PKA, possibly protecting FLNa from proteolysis. The role of FLNa has been well-established in mice lacking FLNa οr in mice expressing a mutant form of GPIbα that does not associate with FLNa [58]. This last model showed that FLNa/GPIbα interaction is absolutely required for platelet adhesion at high pathological shear rates. Furthermore, the study of patients with FLNa mutations (filaminopathy A) also contributed greatly to our knowledge of FLNa function. Though we did confirm that FLNa is required for platelet adhesion on VWF at high shear rates in these patients, we also showed that patients with a missense mutation of FLNa exhibit platelets with a gain-of-adhesion on VWF at pathological and physiological shear rates. These results suggest that FLNa alterations may lead to either thrombosis or hemorrhage. Further studies are required to explore these mechanisms using different models: patient’s platelets but also mutated FLNa in transfected cells.

### Platelet signaling required for αIIbβ3 activation

**Fig. 3:**
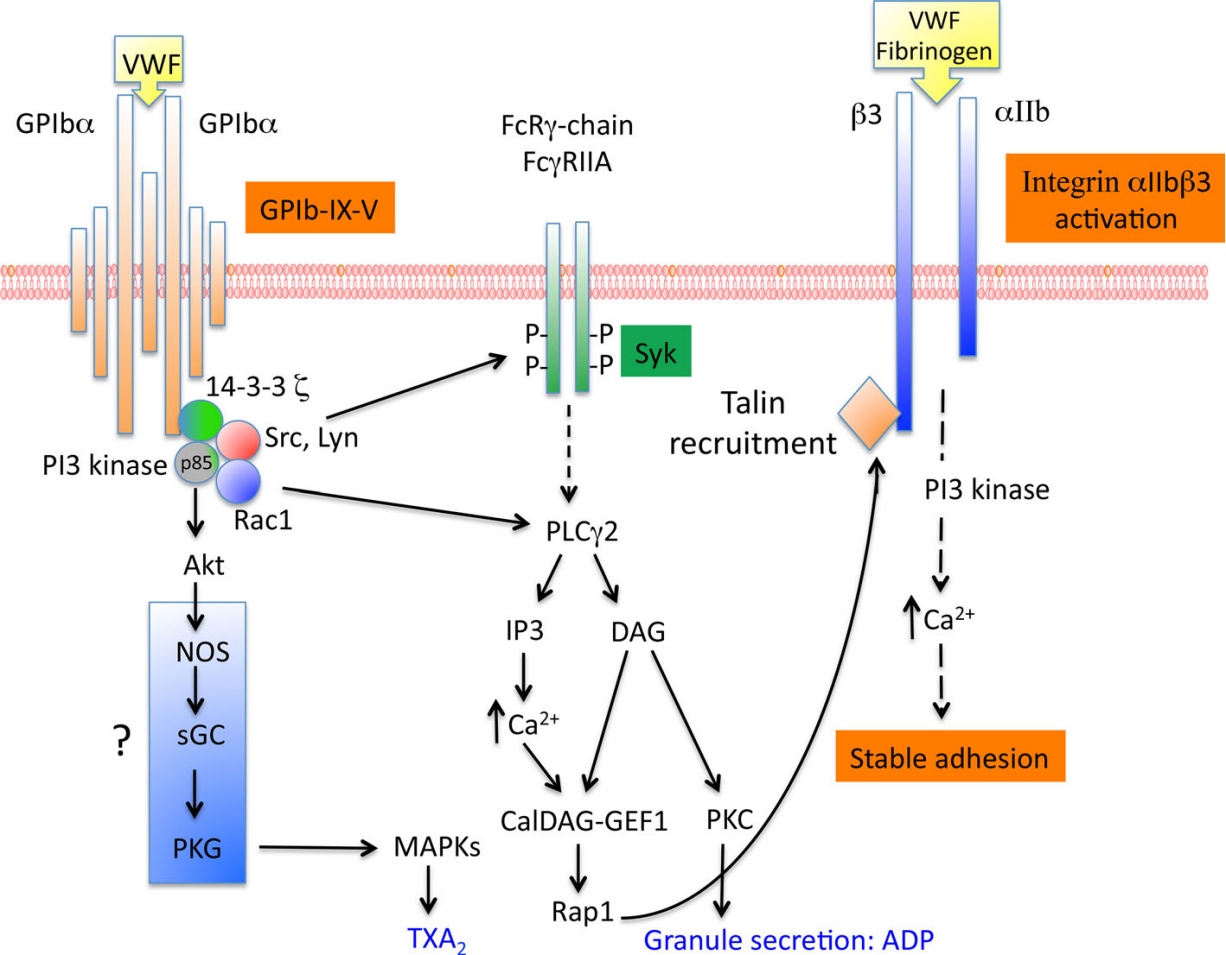
Platelet signaling induced by GPIb-IX-V and required for αIIbβ3 activation. GPIb-IX-V-induced platelet activation is the result of the activation of several molecules. Whereas the involvement of Src family, Rac1, PI3kinase/Akt and the activation of PLCγ1 which leads to Ca^2+^ release is well established, the signaling pathway involving NO and the activation of PKG is still controversial. The subsequent activation of the small G protein Rap1 which allows the recruitment of talin to β3, is essential for αIIbβ3 activation

GPIb-IX-induced platelet activation is a result of the activation of several intracellular molecules including Src family, Rac1, PI3-kinase/Akt, cGMP-dependent protein kinase and MAP kinases. This platelet activation is amplified by the secretion of ADP and by TXA_2_ formation. There is an increasing body of evidence to suggest that lipid rafts provide platforms for the signal transduction pathways related to GPIb-IX-V. Among the signaling pathway cascade involved in αIIbβ3 activation, SFK Lyn and Src have been reported to associate with GPIb-IX after VWF/GPIb-IX activation and to form a complex with PI3-kinase (Fig. 3). The recruitment and activation of Lyn require the p85 subunit of PI3-kinase, which is associated with GPIbα [59]. Lyn is involved in PI3-kinase activation, TXA_2_ formation and secretion during platelet aggregation but also in GPIb-IX mediated integrin-dependent platelet stable adhesion in blood flow independently of TXA_2_ and ADP. In this case, the activation of αIIbβ3 integrin is mediated via the cGMP signaling pathway [60].

Downstream of Lyn, PI3-kinase (which phosphorylates various forms of phosphoinositides in the D3 position of the inositol ring) forms a complex with GPIbα and 14-3-3ζ. The respective associations of 14-3-3ζ and PI3-kinase to GPIbα are independent one from another [61]. PI3-kinase activation is required for αIIbβ3 activation, Ca^2+^ release and thrombus formation in conditions of shear [62]. These results were consolidated later in a murine model where deficiency in PI3-kinase effectors, Akt1 and Akt2 led to impaired GPIb-IX-induced platelet aggregation and stable adhesion under flow [63]. Recently, Rac1 was shown to be involved in GPIb-dependent stable adhesion to VWF under shear stress and its position within the signaling cascade was determined using pharmacological inhibitors and platelet-specific Rac1^−/−^ mice [64]. Indeed, this study clearly showed that Rac1 lies upstream of the PI3-kinase/Akt pathway and downstream of Lyn leading the authors to propose the Lyn/Rac1/PI3-kinase/Akt pathway as mediating VWF-induced activation of αIIbβ3 integrin.

The signaling pathway downstream of PI3-kinase/Akt is still controversial. The team of Du proposed that PI3-kinase/Akt can induce nitric oxide (NO) synthesis, leading to the stimulation of cGMP synthesis [65]. cGMP could then activate PKG, a molecular player involved in platelet secretion required for stabilization of aggregates and in activation of the MAP kinases p38 and ERK [66, 67]. However, other groups have come to contradict these results [68, 69] pointing to a need for further investigations of the NO/cGMP/PKG pathway. Interestingly, we recently participated in a study about a patient with a defect in the α1 subunit of soluble guanylate cyclase (sGC), another member of the NO pathway (NO-sGC-cGMP). We were able to demonstrate that sCG is required for adhesion on VWF in conditions of physiological shear stress confirming the involvement of this pathway in VWF/GPIb induced αIIbβ3 activation [70].

In parallel to the PI3-kinase pathway, another pathway involving Ca^2+^ is also an important actor in αIIbβ3 activation. During platelet adhesion, two waves of Ca^2+^ have been observed. The first wave of Ca^2+^ released from intracellular stores, occuring during platelet translocation, is GPIb-IX-dependent, PI3-kinase-independent and it allows the local activation of αIIbβ3 integrin [71]. This signaling pathway triggered by GPIb involves unknown Src kinase and phospholipase C (PLC) γ2. As for the second sustained wave of Ca^2+^, it is involved in stable adhesion and is dependent on αIIbβ3 integrin and PI3-kinase [71, 72].

### Secondary mediators involved in VWF/GPIb-IX-V signaling

ADP and TXA_2_ are also important secondary mediators. TXA_2_ synthesis can be either dependent or independent of αIIbβ3 activation but the bulk of this synthesis appears to require αIIbβ3 activation [73]. TXA_2_ synthesis is also dependent on the LIM kinase which mediates the activation of PLA_2_ during platelet adhesion [74]. In this case, TXA_2_ synthesis serves as a secondary amplification signaling pathway important in occlusive arterial thrombosis formation but not in the activation of αIIbβ3 integrin. In contrast, ADP via its receptor P2Y1 is required for αIIbβ3 activation and platelet adhesion to subendothelial matrix VWF and through its P2Y12 receptor, for sustained thrombus formation as shown in experiments performed in blood flow [75].

Finally, two receptors with an immunoreceptor tyrosine-based activation motif (ITAM), the Fc receptor γ chain (FcRγ) and the Fcγ receptor IIA (FcγRIIA) have been shown to be associated with GPIb-IX-V [76, 77]. The interaction of VWF with GPIb-IX-V has been reported to lead to tyrosine phosphorylation of 2 ITAM domains of FcRγ and FcγRIIA. However, the importance of these receptors is still debated. Indeed, in platelets treated with anti-FcγRIIA antibodies or in FcRγ-chain deficient platelets, a normal Ca^2+^ mobilization or only slightly reduced Ca^2+^ oscillation and αIIbβ3 activation were observed [78]. Taken together these studies suggest that ITAM receptors are not involved in early activation of αIIbβ3 integrin induced by GPIb-IX-V but they may amplify platelet secretion induced by GPIb-IX-V.

## VWF in adhesion and aggregation: in vitro models

Considerable progress has been made within the last decades in our understanding of the adhesion mechanisms utilized by platelets to adhere to sites of vascular injury. These advances have been achieved in part through improved imaging techniques that enable real-time assessment of platelet thrombus formation “in vitro”. There is now strong evidence that platelets utilize a multistep adhesion mechanism, involving GPIb-IX-V, GPVI and integrin αIIbβ3 to mediate stable adhesion and form aggregates at site of vascular injury. GPIb-IX-V plays a key role in the process of platelet recruitment to the site of vascular injury through specific engagement of the A1 domain of immobilized VWF. This adhesive interaction supports initial platelet tethering and translocation that is insufficient to support firm adhesion. The contribution of new technologies was important to examine the mechanism of translocation on VWF. Indeed, platelets undergo shear-specific morphological changes that may serve to regulate translocation dynamics under flow [79]. Under low shear rates (600 s^−1^), shape change involves extension of membrane tethers and/or filopodia. With increasing shear rates (2,000–5,000 s^−1^), platelets become spheric with numerous surface projections and finally in pathological conditions (10,000–20,000 s^−1^), platelets retract filopodia increasing the rolling velocity. This series of morphological changes involves reorganization of the actin and microtubule cytoskeleton and a signaling pathway dependent on GPIb-IX-V involving Src kinases and Ca^2+^ flux [80]. Firm platelet adhesion represents the second step and involves the collagen receptors α2β1 and GPVI, the fibronectin receptor α2β1 and αIIbβ3 which is the major receptor for VWF and fibrinogen and which is central in the generation of the stable platelet thrombus. The adhesive substrates immobilized on the membrane surface then recruit additional platelets, resulting in aggregation and thrombus formation. These events occur in flowing blood at shear rates exceeding 1,000 s^−1^ in the human circulation. Importantly, this paradigm does not apply under blood flow conditions comparable to those existing in stenotic coronary arteries where shear rates can increase until 20,000–40,000 s^−1^ for example in a coronary artery with a 90 % lumen reduction or just upstream of the stenosis. Ruggeri et al. [81] were the first to report surprising data under such extreme hemodynamic conditions where platelet aggregates can form, exclusively mediated by VWF/GPIb-IX-V interaction without any contribution of activation or αIIbβ3-dependent platelet aggregation. These spectacular new findings showed that VWF/GPIb-IX-V interaction may be a key determinant of platelet accumulation in stenotic arteries leading to acute thrombotic occlusion.

## VWF and thrombus formation: in vivo models

Within the last two decades, mouse engineering has become a powerful tool to explore the mechanisms underlying hemorrhagic and thrombotic disorders. Moreover, the development of various murine experimental thrombosis models, both in arteries and in veins, allowed direct visualization of platelet adhesion and platelet—platelet interaction in real-time. Subsequent quantification of thrombus growth, thrombus stability and formation of emboli provided important new information about the importance of the various molecular players in the kinetics of events leading to the formation of a stable platelet thrombus. While the roles of GPIb-IX-V and VWF in thrombus formation was already well-established in flow chambers studies in conditions of high shear rates, mice deficient for VWF and GPIb-IX-V were essential to precisely determine the contribution of VWF in atherothrombosis and in venous thrombosis in vivo.

### VWF and arterial thrombosis

Mice lacking VWF were the first genetically engineered mice evaluated using an in vivo thrombosis model performed in mesenteric arterioles and visualized through intravital microscopy [13, 82]. Following a ferric-chloride-induced injury of these arterioles, VWF^−/−^ mice exhibited delayed platelet adhesion and reduced thrombus formation. The persistence of an open channel within the thrombus or between thrombus and vascular wall in a large majority of vessels was another characteristic of this model and suggested that VWF plays an essential role in platelet cohesion. Later another model of laser-induced vascular injury in the cremaster muscle microcirculation confirmed these first observations. The absence of VWF did not affect platelet activation, but platelet aggregation and thrombus formation were attenuated [83]. The fact that in mice a defect in VWF leads to a delay in and not to an absence of platelet adhesion and thrombus formation, strongly suggests that other ligands can mediate high shear adhesion in vivo. In addition VWF deficiency in mice appears less severe than a lack of GPIbα [84], suggesting that these alternative adhesive molecules are ligands for GPIbα. Thrombospondin represents a potential candidate as it was shown to mediate GPIbα-dependent platelet adhesion under flow conditions in vitro [85]. Thus, it appears that GPIbα contributes to arterial thrombosis by adhesion mechanisms dependent and independent of VWF. Further studies will be required to explore other candidates potentially involved in platelet adhesion. Finally, the transgenic mouse model expressing the chimeric protein composed of the human IL-4 receptor linked to the cytoplasmic tail of GPIbα clearly showed that GPIbα is absolutely required for recruitment of platelets in thrombi while αIIbβ3 integrin activation is not [84]. This in vivo observation was reminiscent of the GPIbα-dependent but activation-independent thrombus formation in vitro under very high shear rates. With regard to GPIbα, all in vivo thrombosis models, whether the injury was deep or superficial (as differentially obtained using the laser beam model), were consistent with VWF/GPIbα interaction being essential for thrombus formation [86]. In conclusion, GPIbα is essential to the process of arterial thrombosis regardless of the severity of the experimental lesions.

The understanding of the pathophysiological mechanisms of thrombus formation is essential for the design of therapeutic strategies. Atherothrombosis encompasses ischemic stroke which is the second leading cause of death worldwide with 80 % of strokes caused by arterial occlusion of cerebral arteries. Recent work described a role for VWF in ischemic stroke [87, 88]. Indeed, mouse models have shown that the absence of VWF protects mice from brain ischemia. A more precise molecular analysis demonstrated that binding of VWF to both GPIbα and collagen are mandatory steps in stroke development, as opposed to VWF binding to αIIbβ3. The signaling pathway dependent on GPIbα involves phospholipase D1. These observations clearly show that VWF is a critical actor in ischemic stroke and that blocking the VWF/GPIbα axis might be a promising strategy in stroke treatment. In fact, ALX-0081, a nanobody against the A1 domain of VWF that blocks VWF binding to GPIbα, has recently been shown to prevent middle cerebral artery (MCA) thrombosis in guinea pigs and to induce reperfusion when given immediately after complete occlusion, without provoking intracerebral bleeding [89]. These promising data confirm that VWF/GPIbα interaction is essential for platelet adhesion but also for initial thrombus formation in stroke and showed that as opposed to an αIIbβ3 inhibitor (tirofiban), VWF/GPIbα blockade does not lead to hemorrhage. This remarkable observation showed for the first time that a cerebral thrombus can be dissolved by a platelet antagonist. In another recent study, Le Behot et al. [90] confirmed that VWF/GPIbα blockade restored vessel patency after occlusive thrombosis in an MCA model in mice. The authors elegantly showed that an anti-VWF treatment was able to specifically disaggregate the external layer of occlusive thrombi, corresponding to platelet aggregates formed under high shear rates independently of αIIbβ3 integrin activation. These results clearly show that each part of the occlusive thrombi is sensitive to specific thrombolytic agents. To conclude this aspect, inhibitors of VWF/GPIbα interaction currently under preclinical and clinical investigations constitute promising candidates for the treatment of stroke [91].

### VWF and venous thrombosis

While the importance of VWF/GPIbα interaction in arterial thrombosis is well-established and accepted, the role of this interaction in venous thrombosis has long remained unclear with conflicting conclusions from various in vitro models. In the clinical setting, elevated levels of VWF and FVIII have been reported associated with an increased risk of venous thrombosis; however, this effect was due to increased FVIII levels. The contribution of animal models was therefore very important to clarify the exact role of VWF. The first experiments to tackle this issue, a mechanical injury in the hamster femoral vein or a photochemically-induced injury in the mouse jugular vein, relied on the use of inhibitors of VWF and GPIbα. The results showed that the occlusion time was significantly prolonged and that the effect of VWF/GPIbα was dependent on the ERK2 pathway [92, 93]. Next, transgenic mice lacking the GPIbα extracellular domain were studied and showed delayed adhesion in ferric chloride-injured veins but eventually all injured veins occluded. In contrast, in VWF^−/−^ mice, platelet adhesion and thrombus formation were impaired and frequent embolization was observed, the latter being corrected by the infusion of FVIII [94]. This lack of venules occlusion in VWF^−/−^ mice contrasting with the venules occlusion in mice expressing truncated GPIbα strongly suggests that VWF receptors other than GPIbα, probably αΙΙbβ3, are involved in thrombus growth in veins [95]. This important work clearly demonstrates that VWF but also FVIII are essential for the formation of stable occlusive thrombi. This is supported by studies showing that higher levels of plasma VWF due to a single nucleotide polymorphism (rs1063856) in exon eight of VWF are associated with an increase in venous thrombosis risk [96]. Later on, more physiologically relevant models of deep vein thrombosis (DVT) proved essential to further understand the role of platelets and VWF/GPIbα interaction in DVT. Indeed, thrombosis in deep veins is not initiated by a major vessel injury. The challenge was therefore to mimic DVT pathogenesis, i.e. avoiding disruption of the endothelial lining, by using a flow restriction model. VWF-deficient mice were clearly protected from thrombosis induced by complete stasis or partial stenosis flow restriction in the inferior vena cava [97]. In these models, VWF appears to play a critical role in thrombus initiation in the presence of residual blood flow through a mechanism distinct from FVIII-dependent coagulation.

Another link between DVT and VWF also emerged with the discovery of NETs or “neutrophil extracellular traps” [98]. NETs which originate from neutrophils, consist of DNA, citrullinated histones and neutrophil granule constituents. They are implicated in antimicrobial defense and are formed upon vascular injury. NETs contribute to thrombus formation through interaction with platelets and are able to support platelet activation and aggregation suggesting that they can serve as substrates for platelet adhesion. The hypothesis is that adhesive proteins such as VWF could mediate this adhesion. As a support for this hypothesis is a recent work showing that in a model of DVT induced by flow restriction, NETs colocalized with VWF allowing platelet recruitment [99]. Altogether these new findings clearly show that VWF is a component critical in venous thrombosis and that VWF is a potential target for venous thrombosis treatment.

## VWF Regulation: effect on platelet activation

### β2-GPI a natural inhibitor of VWF

The majority of patients with the anti-phospholipid syndrome are characterized by the presence of autoantibodies against the phospholipid-binding protein β2-glycoprotein I (β2-GPI) as well as by the occurrence of thrombotic events, both venous and arterial. Several mechanisms have been described that explain the correlation between the presence of autoantibodies against β2-GPI and these thrombotic complications, one of which is actually related to VWF. The use of a nanobody specific for VWF in its platelet-binding conformation (“active VWF”) showed that the levels of active VWF were significantly increased in patients exhibiting anti-β2-GPI antibodies associated with thrombotic events [100]. Further analysis revealed that β2-GPI selectively recognizes active VWF, and that the formation of β2-GPI/active VWF complexes prevents VWF from interacting with platelets. Thus, β2-GPI seems to act as a natural inhibitor of active VWF, preventing premature VWF-platelet interactions. The presence of autoantibodies specific for β2-GPI suppresses this β2-GPI inhibitory effect, a good explanation for the high levels of circulating active VWF and in turn for the increased thrombotic risk of these patients. The physiological relevance of β2-GPI as a regulator of VWF activity became apparent in a study involving a large cohort of aged men with myocardial infarction [101]. In this study, the authors investigated whether variations in plasma levels of β2-GPI influenced the risk of myocardial infarction. They found a dose-dependent protective effect of increased β2-GPI plasma levels on myocardial infarction in this population. Increased β2-GPI/VWF ratio correlated with a two to three fold reduced risk of myocardial infarction in elderly men. *In vivo* experiments are needed to understand the (patho) physiological role of β2-GPI in hemostasis, and to answer a number of unsettled questions. What is the role of β2-GPI in physiological conditions when VWF is not in its active conformation? Is there any risk of thrombosis in the absence of β2-GPI? Mice lacking β2-GPI do not spontaneously develop thrombosis [102]. Only two unrelated Japanese families with β2-GPI deficiencies have been reported. Neither the patients homozygous for the mutation, nor the heterozygous siblings displayed any thrombotic complications. Though several of their ancestors died of stroke [103], the link with the β2-GPI defect is far from clear, because of concurrent high incidence of hyperlipidemia and diabetes in one family, and because in the other family, the only member reported dead from stroke was 90 years old.

### Regulation of VWF by ADAMTS-13

Thrombotic thrombocytopenic purpura (TTP) is a rare human disease characterized by thrombocytopenia, accumulation of VWF-rich thrombi in the microvasculature, anemia and organ dysfunction. TTP results from a congenital or acquired deficiency in the metalloprotease ADAMTS-13 (A Disintegrin and Metalloprotease with ThromboSpondin type 1 repeat) [104]. Plasma ADAMTS-13 is synthesized and released from hepatic stellate cells and endothelial cells. Its role consists in cleaving VWF multimers within the A2 domain between residues Tyr^1605^-Met^1606^. As a consequence of ADAMTS-13 deficiency, ultra-large (UL) VWF multimers accumulate in the circulation and because of their high biological reactivity, they can spontaneously interact with platelets and trigger intravascular platelet clumps. ADAMTS-13 also cleaves VWF as soon as it is released from endothelial cells, resulting in the shedding of UL-VWF from the endothelial cell surface and in fragmentation of VWF strings [105]. ADAMTS-13 cleavage efficacy is positively regulated by the interaction of VWF with GPIbα under static conditions [106]. Moreover, under fluid shear stress, platelet-VWF complexes increase the proteolytic cleavage of VWF by ADAMTS-13 [107]. These results suggest that the interaction between GPIbα and the VWF A1 domain affects A2 domain accessibility by ADAMTS-13. There appears to exist many levels of regulation of ADAMTS-activity. One example thereof is thrombospondin (TSP-1), which is released from granules following platelet activation triggered by platelet adhesion. TSP-1 shares homology with ADAMTS-13 and like the latter can bind to the A3 domain of VWF. The consequence is that TSP-1 interferes with ADAMTS-13 for binding to UL-VWF multimers therefore inhibiting VWF cleavage and favoring platelet recruitment and thrombus formation [108]. In physiological conditions, plasma ADAMTS-13 is thus essential through its antithrombotic role [109, 110]. Clinical studies have indeed demonstrated that reduced ADAMTS-13 activity and increased VWF levels are risk factors for the development of myocardial infarction and ischemic stroke [111, 112]. Recently, Casari et al. [113] developed an original mouse model of TTP expressing a mutant VWF resistant to proteolysis. This model should prove interesting in the study of the role of continuous platelet aggregation in the context of thrombosis. Furthermore, this model based on the interaction of VWF with GPIbα will be useful to study molecular events associated with the transition from low to high platelet activation.

## Pathologies of VWF and platelet functions

The fundamental role of VWF in hemostasis is illustrated by the bleeding tendency of patients suffering from von Willebrand disease (VWD). This bleeding disorder is classified into three major types which reflect the clinical heterogeneity of this disease. VWD-type 1 is characterized by a partial quantitative deficiency of VWF and represents the most common form of VWD (70–80 %). Large population studies showed that there is a gradient of increasing influence of pathogenic *VWF* mutations [114–117]. Clinical bleeding history, reduction of plasma VWF and often family history characterize VWD-type 1. In contrast to VWD-type 1, VWD-type 3 is due to a severe quantitative deficiency in VWF with a major reduction in FVIII and represents less than 1 % of VWD cases. VWD-type 2 is characterized by a qualitative deficiency in VWF and accounts for approximately 20 % of all cases of VWD. VWD-type 2 is sub-divided into 4 variants: 2A, 2B, 2 M and 2 N. VWD-type 2 N mutations result in decreased FVIII binding to VWF. VWF mutations in VWD-types 2A and 2 M result in reduced platelet binding capability. In contrast, VWD-type 2B corresponds to enhanced VWF-platelet binding capability. In this review, we will address various aspects of the regulation of VWF and its impact on platelet activation will also be approached.

### VWF mutations resulting in reduced platelet binding capability

Mutations in VWD-types 2A and 2 M lead to reduced VWF/platelet interaction. In VWD-type 2A, high molecular weight (HMW) VWF multimers are lost. More than 70 different mutations have been described, located in the A1 or A2 domains of VWF. A1 domain mutations resulting in VWD-type 2A affect the normal protein structure, interfering with biosynthesis, multimer assembly, storage and release [118]. Other VWD-type 2A mutations, located in the A2 domain, enhance VWF susceptibility to ADAMTS-13-mediated proteolysis [119]. The bleeding symptoms are clearly explained by defective ristocetin-induced platelet aggregation in an aggregometer [120]. To explore the mechanisms underlying the bleeding phenotype, Sugimoto et al. [121] analyzed thrombus formation using blood from patients with VWD-type 2A on a collagen matrix under flow conditions. They were able to show that thrombus formation was impaired at high shear rates and the defect became more prominent with increasing shear rates, reflecting the lack of HMWs of VWF.

VWD-type 2 M is characterized by a loss-of-function but in contrast to type 2A, HMWs are present with a normal multimer distribution. The VWF binding capacity of platelets is reduced due to modifications in the A1 domain structure and configuration [122]. Most of the point mutations are located in the A1 domain where they directly affect VWF/GPIbα interaction [123, 124]. Several reports have shown that VWD-type 2 M can also result from mutations affecting VWF binding to collagen. Such mutations can be located either in the A1 or A3 domain [123, 125–127]. Binding of VWF to fibrillar collagens (type I and III) is primarily mediated by the A3 domain but which under specific circumstances, can be substituted for by the A1 domain [128]. In contrast, the A1 domain is the most prominent domain involved in VWF binding to microfibrillar collagen type VI [129]. Recent evidence shows that VWD-type 2 M is associated with a bleeding phenotype milder than most other types of VWD [130]. In conclusion, VWD-type 2 M represents a spectrum of functional defects, including altered platelet binding, altered collagen binding or both. More studies are needed to explain the impact of a defect in collagen binding on the clinical bleeding phenotype. In VWD subtypes 2A and 2 M as well as in the 2 N subtype, no platelet defect has ever been reported.

### VWF mutations resulting in enhanced platelet binding capability

**Fig. 4:**
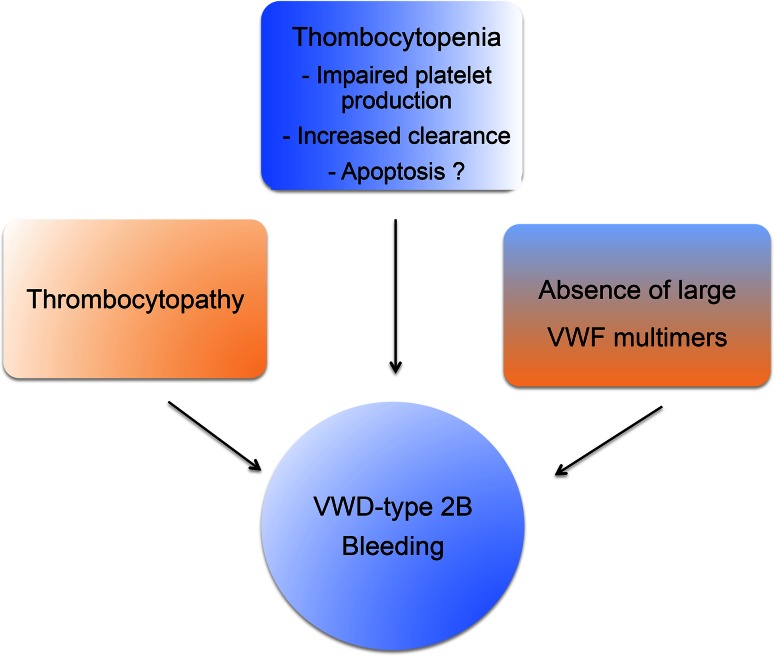
VWD-type 2B and bleeding phenotype. VWD-type 2B and its bleeding phenotype result from a combination of pathological mechanisms involving absence of large VWF multimers, thrombocytopathy and thrombocytopenia

VWD-type 2B (5–8 % of all VWD) is characterized by a gain-of-platelet function and surprisingly enough by a bleeding phenotype. The bleeding phenotype in VWD-type 2B is often explained by 1) the unavailability of GPIbα due to constitutively bound VWF 2B mutant 2) the absence of HMW-VWF multimers, the most functionally effective forms of VWF and 3) the moderate-to-severe thrombocytopenia observed in these patients (Fig. 4). VWD-type 2B mutations induce a gain-of-function characterized by an increased affinity for platelet glycoprotein GPIbα [131] leading to enhanced ristocetin-induced platelet agglutination and to spontaneous platelet agglutination in vitro and in vivo. These mutations are located in exon 28 coding for the A1 domain of the *VWF* gene. More than 20 mutations have been listed. VWD-type 2B is also characterized by the disappearance of HMW VWF in plasma despite a normal synthesis. Although the basic bleeding phenotype of VWD-type 2B can be accounted for by the constitutive binding of mutant VWF 2B to platelets, the platelet defects described in various reports appear puzzling and require further attention. For example, ultrastructural morphological abnormalities of platelets, including giant platelets, are common in VWD-type 2B [132–135]. Another characteristic of VWD-type 2B is the thrombocytopenia the origin, variability and fluctuation of which remain unclear. In a large study enrolling 67 VWD-type 2B patients, it was clearly shown that the variable degree of thrombocytopenia is mutation-dependent and that the bleeding tendency is directly correlated with platelet counts [136]. Different hypotheses, not mutually exclusive, could explain thrombocytopenia in VWD-type 2B. The first is that thrombocytopenia may originate from impaired platelet production. In normal circumstances, the VWF/GPIb interaction has a positive influence on proplatelet formation from MKs in culture, as shown by Nurden et al. [137]. However, the presence of VWF carrying a VWD-type 2B mutation has deleterious effects on MKs exhibiting limited extension of pseudopodia and structure abnormalities in platelets with few large proplatelets. However, impaired megakaryopoiesis alone may not completely explain thrombocytopenia. The second possibility is that thrombocytopenia may be explained by the presence of circulating VWF/platelet aggregates (secondary to spontaneous platelet binding of mutant VWF), undergoing clearance from plasma. This hypothesis is supported by the observation that VWF is present at the platelet surface in VWD-type 2B patients [132] and by our own experimental in vivo results. Our group has indeed generated a mouse model of VWD-type 2B (based on hydrodynamic injection of a p.V1316M VWF-expressing plasmid) which reproduces the clinical phenotype of human patients with a severe bleeding phenotype, thrombocytopenia, circulating aggregates and giant platelets [138]. We found that VWF was detectable at the surface of platelets only in mice injected with the p.V1316M/VWF-expressing plasmid, and not in control mice injected with the wild type VWF-expressing plasmid [139]. Using this model, we were able to show that VWF/platelet aggregates are removed by liver and spleen macrophages and that as a consequence, VWF-type 2B platelets had a circulatory half-life shorter than wild type platelets, therefore contributing to the lower platelet count [139]. It should be noted that this process is independent of VWF multimer size, meaning that HMW multimers are not the only VWF species absorbed onto platelets. The lack of HMW multimers in VWD-type 2B is caused by an increased susceptibility of mutant VWF to ADAMTS-13 [138].

A third potential mechanism for thrombocytopenia in VWF-type 2B is platelet apoptosis. Indeed, VWF-GPIbα interaction has been reported to induce formation of apoptotic platelets via a pathway involving caspase 3 and the proapoptotic proteins Bak and Bax [140]. In addition, cold-storage of platelets followed by rewarming, triggers apoptosis through 14-3-3ζ association with GPIbα and dissociation from the proapoptotic protein Bad allowing its activation [141]. However, our preliminary experiments on our mouse model for VWF-type 2B and on platelets from a VWD-type 2B patient exhibiting a severe thrombocytopenia did not show any apoptosis (Berrou et al., unpublished data). We thus cannot conclude as to apoptosis being involved in thrombocytopenia, at least in the patient studied, but additional studies are needed to explore this issue in more details. In conclusion, the current view for the bleeding phenotype in VWD-type 2B explained on one hand by the absence of the highly hemostatic HMW VWF multimers and on the other hand by thrombocytopenia remains valid. Our work has contributed to the explanation at least partially of the mechanisms behind these two observations: increased ADAMTS-13 proteolysis for the lack of HMW multimers and enhanced phagocytosis of mutant VWF-bound platelets by macrophages for the thrombocytopenia.

**Fig. 5:**
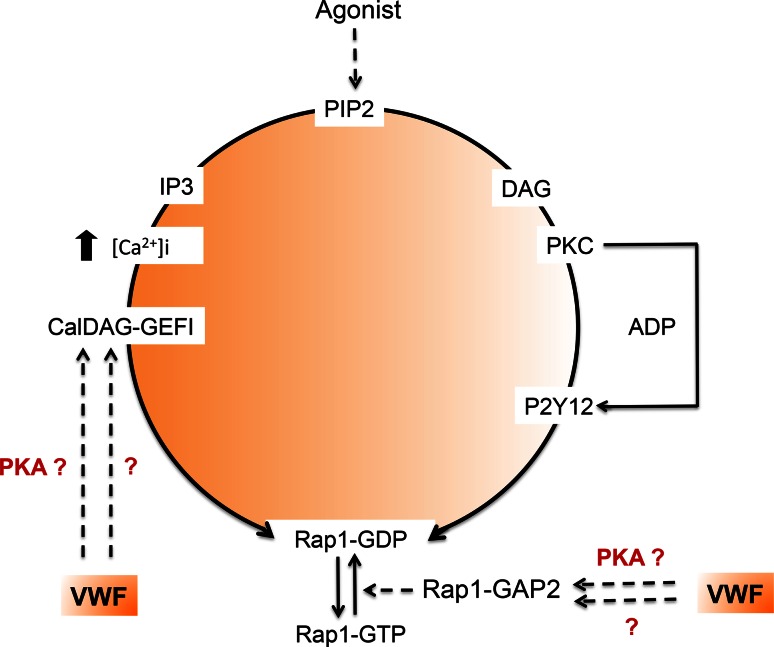
VWD-type 2B and activation of the small G protein Rap1. Platelet aggregation, secretion and αIIbβ3 activation induced by various agonists are impaired in the presence of VWF/p.V1316M. Ca^2+^ mobilization required for αIIbβ3 activation is normal, whereas, the activation of Rap1 required for talin recruitment is impaired. Rap1 activity is controlled by the guanidine nucleotide exchange

However, another question is raised by VWD-type 2B: could the inappropriate constitutive binding of VWF to platelets alter platelet function and hence contribute to the bleeding phenotype? Indeed, the low platelet count alone does not convincingly explain the bleeding tendency. A recent study showed that mice with platelet counts as low as 10 % of controls displayed normal hemostasis. Even considering the limitations of the mouse model in hemostasis, this suggests that thrombocytopenia is probably not the only cause of the bleeding phenotype observed in VWD-type 2B [142]. The observation of giant platelets in some VWD-type 2B patients is the first hint of platelet dysfunction in VWD. A more direct functional impact of mutant VWF 2B on platelets has been partially addressed in the past. The first study describing a defect in platelet functions in VWD-type 2B comes from a patient exhibiting the so-called Montreal Platelet Syndrome [143] which 25 years later was shown to correspond to VWD-type 2B with the p.V1316M mutation [144]. Montreal Platelet Syndrome is characterized by mucocutaneous bleeding, thrombocytopenia with large platelets, spontaneous platelet aggregates in plasma and by an unexpected defect in platelet aggregation induced by thrombin [144]. Two other observations reported VWD-type 2B patients with reduced platelet aggregation and secretion correlating with impaired granule content [134] as well as a heterogeneous defect in thrombus growth using perfusion assays on collagen matrix [121]. Using three different models, platelets from mice expressing the VWF/p.V1316M mutation, a patient harboring the same mutation and control platelets in the presence of recombinant VWF/p.V1316M, we clearly found that platelet aggregation, platelet secretion, and integrin αIIbβ3 activation were impaired, whatever the agonist [145]. Altered platelet functions correlated with impaired thrombus formation. Ca^2+^ mobilization which is required for αIIbβ3 activation was normal. In contrast, the activation of the small G protein Rap1, which is required for talin recruitment by integrin αIIbβ3 and its subsequent activation, was impaired [145]. Our conclusion is that VWF/p.V1316M acts on the activation pathway of Rap1 but downstream of Ca^2+^ mobilization. Rap1 activity is controlled both by the guanidine nucleotide exchange factor (GEF) CalDAG-GEF1 that stimulates the release of GDP and the binding of GTP to Rap1, as well as by the GTPase-activating proteins that stimulate the intrinsic Rap1 GTPase activity (Fig. 5) [146, 147]. Recently, CalDAG-GEF1 was shown to be negatively regulated by PKA, an inhibitor of platelet activation [148]. Furthermore, Rap1Gap2, the only known GAP of Rap1 in platelets [149] was also shown to be regulated by PKA and PKG [150]. It is thus possible that VWD-type 2B VWF binding to GPIb elicits activation of PKA or PKG which would then block the Rap1-αIIbβ3 pathway. However, further studies are required to test this hypothesis.

Clearly, the VWD-type 2B mutation p.V1316M is associated with a severe thrombocytopathy which very likely contributes to the bleeding tendency in VWD-type 2B. Several questions still remain without answers. The signaling pathway induced by VWF/p.V1316M is unknown and requires more detailed investigation. Preliminary data show that in control platelets recombinant VWF/p.V1316M induces a specific signaling pathway involving activation of phosphatases (Berrou et al., unpublished results). Further studies will be required to investigate the role of these phosphatases in the VWD-type 2B-associated thrombopathy. The other possibility is that intrinsic signaling defects in VWF-type 2B platelets are a consequence of altered platelet biogenesis by megakaryocytes. This hypothesis is supported by the fact that VWF-type 2B platelets but not control platelets (in the presence of recombinant VWF/pV1316M), exhibited defective Ca^2+^ influx, which is likely to affect platelet activation. Other questions may be: are structural changes of mutated VWF and/or GPIb-IX-V required or not for their spontaneous interaction? Is the severe bleeding tendency exclusively due to the thrombocytopathy or not? Do mutations other than V1316M exhibit the same platelet functional inhibition? Finally, Jerry Ware raised the issue of potential implications for anti-thrombotic targeting of VWF/GPIb-IX axis [151]. Indeed, could type 2B mimetics bind to normal platelets and inhibit αIIbβ3 activation, thereby initiating a new strategy for antithrombotic therapy? Obviously this will require thorough future investigations. Finally, this study suggests that for a better evaluation of the bleeding tendency associated with VWD-type 2B, investigations of platelet functions must be considered in the clinical assessment of the disease.

### Platelet mutations resulting in enhanced binding of VWF

Platelet-type von Willebrand disease (Pt-VWD) also called pseudo-von Willebrand disease, is a gain-of-functions condition similar to VWD-type 2B characterized by spontaneous binding of plasma VWF to platelets and increased platelet agglutination with low amounts of ristocetin. Pt-VWD is caused by *GPIBA* mutations with autosomal dominant inheritance and is associated with a moderate thrombocytopenia [152, 153]. The mutations in the extracellular domain of GPIbα lead to increased affinity of VWF for GPIb-IX-V and to an increased bleeding risk [154, 155]. This phenotype appears to be significantly less frequent than VWD-type 2B. Only 4 GPIbα mutations have been described so far [154–157]. A mouse model expressing a mutant human subunit GPIbα (GPIbα/PG233 V) associated with Pt-VWD displays several diagnostic attributes of Pt-VWD, including the ability of platelets to agglutinate at low doses of ristocetin and a bleeding phenotype [158]. In vivo, a complete abrogation of experimentally-induced thrombosis of the carotid artery in a mouse model of Pt-VWD was observed [159]. Interestingly and like in VWD-type 2B, impaired platelet functions induced by various agonists such as ADP and thrombin were also observed in Pt-VWF [159]. This confirms that abnormal signaling induced by inappropriate VWF/GPIb interaction and/or abnormal platelet biogenesis by megakaryocytes can lead to impaired platelet functions in VWF-type 2B and in Pt-VWF [145]. The abnormal signaling in Pt-VWD remains to be identified.

In conclusion, the gain-of-platelet functions observed in VWD-type 2B and Pt-VWF clearly lead to abnormal signaling leading to thrombocytopathy. Identification of the signaling pathways involved may lead to a new anti-thrombotic strategy.

## Conclusions and perspectives

In the last three decades, the classical role of VWF in hemostasis has largely been studied. Within the last years, a substantial amount of data supporting the role of VWF in thrombosis has emerged. The development of genetically engineered mice has confirmed the crucial role of VWF in arterial thrombosis but also in venous thrombosis. Moreover, the development of specific murine experimental models of pathological thrombosis that mimic human vascular disease (DVT, stroke) was a great advance in the understanding of the mechanisms and in designing new therapeutic strategies. The inhibitors of VWF/GPIbα interaction which are currently under preclinical and clinical investigations appear as promising candidates for the treatment of stroke while keeping the bleeding risk low [91]. Another future alternative strategy to decrease thrombus without undermining hemostasis is the targeting of the signaling pathways involved in platelet activation. Preclinical studies have demonstrated that pharmacological inhibitors of the PI3-kinase β involved in GPIb-IX-V signaling are effective at preventing thrombotic occlusion of arteries and suggest that these inhibitors may cause less bleeding than conventional approaches [160]. The challenge of the next years will be to establish future strategies for a safe combination of anti-thrombotic therapies.

In parallel to the development of new models of thrombosis, many aspects of the molecular biology of VWF remain to be explored. Recent investigations have revealed that mutated VWF in VWD-type 2B is able to prevent platelet aggregation and consequently to induce a thrombocytopathy, explaining in part the bleeding tendency observed in these patients. The identification of the signaling pathways induced by mutated VWF is open and constitutes an important new goal which may lead to a new anti-thrombotic strategy. This new challenge requires the investigation of different mutated VWF to assess their effect on platelet aggregation as well as the signaling pathways involved in relation with the clinical situation. These new investigations must now be considered in the clinical assessment of the disease and should be clinically useful for predicting the severity of bleeding in patients with VWD-type 2B.

Another important point yet to be resolved is that the signaling pathways associated with thrombocytopathies involving the VWF-GPIbα axis are not fully characterized. Giant platelets observed in Bernard Soulier syndrome, VWD-type 2B and recently in filaminopathy A strongly suggest that the VWF/GPIbα/FLNa axis is essential to maintain platelet shape and for proplatelet formation. Recent evidence showed that VWF/GPIbα interaction is able to induce a pro-apoptotic signaling pathway in platelets. Although very attractive to explain thrombocytopenia in VWD-type 2B, this hypothesis was unfortunately not confirmed by our preliminary experiments on VWD-type 2B platelets but more patients need to be tested. The complexity of this hypothesis is that apoptotic platelets can be rapidly eliminated from the circulation, thus preventing their detection, raising the question of the feasibility of the identification of such an apoptotic pathway. The other possibility is that in VWD-type 2B, apoptosis occurs not in platelets, but in MKs which regulate proplatelet formation. Further work will be required to elucidate the signaling pathways associated with thrombocytopathies in VWD-type 2B.

Finally, in the circulation, recent discoveries showed that besides factor VIII, other proteins (osteoprotegerin, angiopoietin, ADAMTS-13) are associated with VWF in the circulation. One of the new challenges in the next years will be to examine whether these associations may affect the interaction between VWF and its platelet ligands GPIb-IX-V and integrin αIIbβ3.

## References

[1] Savage B, Almus-Jacobs F, Ruggeri ZM (1998). Specific synergy of multiple substrate-receptor interactions in platelet thrombus formation under flow. Cell.

[2] Ginsburg D, Handin RI, Bonthron DT, Donlon TA, Bruns GA, Latt SA, Orkin SH (1985). Human von Willebrand factor (vWF): isolation of complementary DNA (cDNA) clones and chromosomal localization. Science.

[3] Goodeve AC (2010). The genetic basis of von Willebrand disease. Blood Rev.

[4] ZhouYF Eng ET, Zhu J, Lu C, Walz T, Springer TA (2012). Sequence and structure relationships within von Willebrand factor. Blood.

[5] Katsumi A, Tuley EA, Bodo I, Sadler JE (2000). Localization of disulfide bonds in the cystine knot domain of human von Willebrand factor. J Biol Chem.

[6] Mayadas TN, Wagner DD (1989). In vitro multimerization of von Willebrand factor is triggered by low pH. Importance of the propolypeptide and free sulfhydryls. J Biol Chem.

[7] van de Ven WJ, Voorberg J, Fontijn R, Pannekoek H, van den Ouweland AM, van Duijnhoven HL, Roebroek AJ, Siezen RJ (1990). Furin is a subtilisin-like proprotein processing enzyme in higher eukaryotes. Mol Biol Rep.

[8] Canis K, McKinnon TA, Nowak A, Haslam SM, Panico M, Morris HR, Laffan MA, Dell A (2012). Mapping the *N*-glycome of human von Willebrand factor. Biochem J.

[9] Canis K, McKinnon TA, Nowak A, Panico M, Morris HR, Laffan M, Dell A (2010). The plasma von Willebrand factor O-glycome comprises a surprising variety of structures including ABH antigens and disialosyl motifs. J Thromb Haemost.

[10] Matsui T, Titani K, Mizuochi T (1992). Structures of the asparagine-linked oligosaccharide chains of human von Willebrand factor. Occurrence of blood group A, B, and H(O) structures. J Biol Chem.

[11] Wagner DD, Olmsted JB, Marder VJ (1982). Immunolocalization of von Willebrand protein in Weibel-Palade bodies of human endothelial cells. J Cell Biol.

[12] Sporn LA, Chavin SI, Marder VJ, Wagner DD (1985). Biosynthesis of von Willebrand protein by human megakaryocytes. J Clin Invest.

[13] Denis C, Methia N, Frenette PS, Rayburn H, Ullman-Cullere M, Hynes RO, Wagner DD (1998). A mouse model of severe von Willebrand disease: defects in hemostasis and thrombosis. Proc Natl Acad Sci USA.

[14] Huang RH, Wang Y, Roth R, Yu X, Purvis AR, Heuser JE, Egelman EH, Sadler JE (2008). Assembly of Weibel-Palade body-like tubules from N-terminal domains of von Willebrand factor. Proc Natl Acad Sci USA.

[15] Zhou YF, Eng ET, Nishida N, Lu C, Walz T, Springer TA (2011). A pH-regulated dimeric bouquet in the structure of von Willebrand factor. EMBO J.

[16] Valentijn KM, Sadler JE, Valentijn JA, Voorberg J, Eikenboom J (2011). Functional architecture of Weibel-Palade bodies. Blood.

[17] van Breevoort D, van Agtmaal EL, Dragt BS, Gebbinck JK, Dienava-Verdoold I, Kragt A, Bierings R, Horrevoets AJ, Valentijn KM, Eikenboom JC, Fernandez-Borja M, Meijer AB, Voorberg J (2012). Proteomic screen identifies IGFBP7 as a novel component of endothelial cell-specific Weibel-Palade bodies. J Proteome Res.

[18] Fiedler U, Scharpfenecker M, Koidl S, Hegen A, Grunow V, Schmidt JM, Kriz W, Thurston G, Augustin HG (2004). The Tie-2 ligand angiopoietin-2 is stored in and rapidly released upon stimulation from endothelial cell Weibel-Palade bodies. Blood.

[19] Italiano JE, Battinelli EM (2009). Selective sorting of alpha-granule proteins. J Thromb Haemost.

[20] Nightingale T, Cutler D (2013). The secretion of von Willebrand factor from endothelial cells; an increasingly complicated story. J Thromb Haemost.

[21] Romani de Wit T, Rondaij MG, Hordijk PL, Voorberg J, van Mourik JA (2003). Real-time imaging of the dynamics and secretory behavior of Weibel-Palade bodies. Arterioscler Thromb Vasc Biol.

[22] Andre P, Denis CV, Ware J, Saffaripour S, Hynes RO, Ruggeri ZM, Wagner DD (2000). Platelets adhere to and translocate on von Willebrand factor presented by endothelium in stimulated veins. Blood.

[23] Valentijn KM, van Driel LF, Mourik MJ, Hendriks GJ, Arends TJ, Koster AJ, Valentijn JA (2010). Multigranular exocytosis of Weibel-Palade bodies in vascular endothelial cells. Blood.

[24] Torisu T, Torisu K, Lee IH, Liu J, Malide D, Combs CA, Wu XS, Rovira II, Fergusson MM, Weigert R, Connelly PS, Daniels MP, Komatsu M, Cao L, Finkel T (2013). Autophagy regulates endothelial cell processing, maturation and secretion of von Willebrand factor. Nat Med.

[25] Goudemand J, Scharrer I, Berntorp E, Lee CA, Borel-Derlon A, Stieltjes N, Caron C, Scherrmann JM, Bridey F, Tellier Z, Federici AB, Mannucci PM (2005). Pharmacokinetic studies on Wilfactin, a von Willebrand factor concentrate with a low factor VIII content treated with three virus-inactivation/removal methods. J Thromb Haemost.

[26] Brown SA, Eldridge A, Collins PW, Bowen DJ (2003). Increased clearance of von Willebrand factor antigen post-DDAVP in type 1 von Willebrand disease: is it a potential pathogenic process?. J Thromb Haemost.

[27] Sztukowska M, Gallinaro L, Cattini MG, Pontara E, Sartorello F, Daidone V, Padrini R, Pagnan A, Casonato A (2008). Von Willebrand factor propeptide makes it easy to identify the shorter Von Willebrand factor survival in patients with type 1 and type Vicenza von Willebrand disease. Br J Haematol.

[28] Gallinaro L, Cattini MG, Sztukowska M, Padrini R, Sartorello F, Pontara E, Bertomoro A, Daidone V, Pagnan A, Casonato A (2008). A shorter von Willebrand factor survival in O blood group subjects explains how ABO determinants influence plasma von Willebrand factor. Blood.

[29] Gill JC, Endres-Brooks J, Bauer PJ, Marks WJ, Montgomery RR (1987). The effect of ABO blood group on the diagnosis of von Willebrand disease. Blood.

[30] Casari C, Lenting PJ, Wohner N, Christophe OD, Denis CV (2013). Clearance of von Willebrand factor. J Thromb Haemost.

[31] van Schooten CJ, Shahbazi S, Groot E, Oortwijn BD, van den Berg HM, Denis CV, Lenting PJ (2008). Macrophages contribute to the cellular uptake of von Willebrand factor and factor VIII in vivo. Blood.

[32] Grewal PK, Uchiyama S, Ditto D, Varki N, Le DT, Nizet V, Marth JD (2008). The Ashwell receptor mitigates the lethal coagulopathy of sepsis. Nat Med.

[33] Pegon JN, Kurdi M, Casari C, Odouard S, Denis CV, Christophe OD, Lenting PJ (2012). Factor VIII and von Willebrand factor are ligands for the carbohydrate-receptor Siglec-5. Haematologica.

[34] Rydz N, Swystun LL, Notley C, Paterson AD, Riches JJ, Sponagle K, Boonyawat B, Montgomery RR, James PD, Lillicrap D (2013). The C-type lectin receptor CLEC4 M binds, internalizes, and clears von Willebrand factor and contributes to the variation in plasma von Willebrand factor levels. Blood.

[35] Rastegarlari G, Pegon JN, Casari C, Odouard S, Navarrete AM, Saint-Lu N, van Vlijmen BJ, Legendre P, Christophe OD, Denis CV, Lenting PJ (2012). Macrophage LRP1 contributes to the clearance of von Willebrand factor. Blood.

[36] Lenting PJ, Neels JG, van den Berg BM, Clijsters PP, Meijerman DW, Pannekoek H, van Mourik JA, Mertens K, van Zonneveld AJ (1999). The light chain of factor VIII comprises a binding site for low density lipoprotein receptor-related protein. J Biol Chem.

[37] Morange PE, Tregouet DA, Frere C, Saut N, Pellegrina L, Alessi MC, Visvikis S, Tiret L, Juhan-Vague I (2005). Biological and genetic factors influencing plasma factor VIII levels in a healthy family population: results from the Stanislas cohort. Br J Haematol.

[38] Castro-Nunez L, Dienava-Verdoold I, Herczenik E, Mertens K, Meijer AB (2012). Shear stress is required for the endocytic uptake of the factor VIII-von Willebrand factor complex by macrophages. J Thromb Haemost.

[39] Ramakrishnan V, Reeves PS, DeGuzman F, Deshpande U, Ministri-Madrid K, DuBridge RB, Phillips DR (1999). Increased thrombin responsiveness in platelets from mice lacking glycoprotein V. Proc Natl Acad Sci USA.

[40] Kahn ML, Diacovo TG, Bainton DF, Lanza F, Trejo J, Coughlin SR (1999). Glycoprotein V-deficient platelets have undiminished thrombin responsiveness and Do not exhibit a Bernard-Soulier phenotype. Blood.

[41] Moog S, Mangin P, Lenain N, Strassel C, Ravanat C, Schuhler S, Freund M, Santer M, Kahn M, Nieswandt B, Gachet C, Cazenave JP, Lanza F (2001). Platelet glycoprotein V binds to collagen and participates in platelet adhesion and aggregation. Blood.

[42] Gardiner EE, Karunakaran D, Shen Y, Arthur JF, Andrews RK, Berndt MC (2007). Controlled shedding of platelet glycoprotein (GP)VI and GPIb-IX-V by ADAM family metalloproteinases. J Thromb Haemost.

[43] Berndt MC, Metharom P, Andrews RK (2014). Primary haemostasis: newer insights. Haemophilia.

[44] Nurden A, Nurden P (2011). Advances in our understanding of the molecular basis of disorders of platelet function. J Thromb Haemost.

[45] Zimmerman TS, Ruggeri ZM (1982). Von Willebrand’s disease. Prog Hemost Thromb.

[46] Lopez JA, Andrews RK, Afshar-Kharghan V, Berndt MC (1998). Bernard-Soulier syndrome. Blood.

[47] Ware J, Russell SR, Marchese P, Murata M, Mazzucato M, De Marco L, Ruggeri ZM (1993). Point mutation in a leucine-rich repeat of platelet glycoprotein Ib alpha resulting in the Bernard-Soulier syndrome. J Clin Invest..

[48] Kanaji T, Russell S, Ware J (2002). Amelioration of the macrothrombocytopenia associated with the murine Bernard-Soulier syndrome. Blood.

[49] Falet H, Pollitt AY, Begonja AJ, Weber SE, Duerschmied D, Wagner DD, Watson SP, Hartwig JH (2010). A novel interaction between FlnA and Syk regulates platelet ITAM-mediated receptor signaling and function. J Exp Med.

[50] Nurden P, Debili N, Coupry I, Bryckaert M, Youlyouz-Marfak I, Sole G, Pons AC, Berrou E, Adam F, Kauskot A, Lamaziere JM, Rameau P, Fergelot P, Rooryck C, Cailley D, Arveiler B, Lacombe D, Vainchenker W, Nurden A, Goizet C (2011). Thrombocytopenia resulting from mutations in filamin A can be expressed as an isolated syndrome. Blood.

[51] Berrou E, Adam F, Lebret M, Fergelot P, Kauskot A, Coupry I, Jandrot-Perrus M, Nurden A, Favier R, Rosa JP, Goizet C, Nurden P, Bryckaert M (2013). Heterogeneity of platelet functional alterations in patients with filamin A mutations. Arterioscler Thromb Vasc Biol.

[52] Nurden AT (2006). Glanzmann thrombasthenia. Orphanet J Rare Dis.

[53] Hodivala-Dilke KM, McHugh KP, Tsakiris DA, Rayburn H, Crowley D, Ullman-Cullere M, Ross FP, Coller BS, Teitelbaum S, Hynes RO (1999). Beta3-integrin-deficient mice are a model for Glanzmann thrombasthenia showing placental defects and reduced survival. J Clin Invest.

[54] Mangin P, David T, Lavaud V, Cranmer SL, Pikovski I, Jackson SP, Berndt MC, Cazenave JP, Gachet C, Lanza F (2004). Identification of a novel 14-3-3zeta binding site within the cytoplasmic tail of platelet glycoprotein Ibalpha. Blood.

[55] Bodnar RJ, Xi X, Li Z, Berndt MC, Du X (2002). Regulation of glycoprotein Ib-IX-von Willebrand factor interaction by cAMP-dependent protein kinase-mediated phosphorylation at Ser 166 of glycoprotein Ib(beta). J Biol Chem.

[56] Dai K, Bodnar R, Berndt MC, Du X (2005). A critical role for 14-3-3zeta protein in regulating the VWF binding function of platelet glycoprotein Ib-IX and its therapeutic implications. Blood.

[57] Du X (2007). Signaling and regulation of the platelet glycoprotein Ib-IX-V complex. Curr Opin Hematol.

[58] Cranmer SL, Ashworth KJ, Yao Y, Berndt MC, Ruggeri ZM, Andrews RK, Jackson SP (2011). High shear-dependent loss of membrane integrity and defective platelet adhesion following disruption of the GPIbalpha-filamin interaction. Blood.

[59] Wu Y, Asazuma N, Satoh K, Yatomi Y, Takafuta T, Berndt MC, Ozaki Y (2003). Interaction between von Willebrand factor and glycoprotein Ib activates Src kinase in human platelets: role of phosphoinositide 3-kinase. Blood.

[60] Yin H, Liu J, Li Z, Berndt MC, Lowell CA, Du X (2008). Src family tyrosine kinase Lyn mediates VWF/GPIb-IX-induced platelet activation via the cGMP signaling pathway. Blood.

[61] Mu FT, Andrews RK, Arthur JF, Munday AD, Cranmer SL, Jackson SP, Stomski FC, Lopez AF, Berndt MC (2008). A functional 14-3-3zeta-independent association of PI3-kinase with glycoprotein Ib alpha, the major ligand-binding subunit of the platelet glycoprotein Ib-IX-V complex. Blood.

[62] Yap CL, Anderson KE, Hughan SC, Dopheide SM, Salem HH, Jackson SP (2002). Essential role for phosphoinositide 3-kinase in shear-dependent signaling between platelet glycoprotein Ib/V/IX and integrin alpha(IIb)beta(3). Blood.

[63] Yin H, Stojanovic A, Hay N, Du X (2008). The role of Akt in the signaling pathway of the glycoprotein Ib-IX induced platelet activation. Blood.

[64] Delaney MK, Liu J, Zheng Y, Berndt MC, Du X (2012). The role of Rac1 in glycoprotein Ib-IX-mediated signal transduction and integrin activation. Arterioscler Thromb Vasc Biol.

[65] Stojanovic A, Marjanovic JA, Brovkovych VM, Peng X, Hay N, Skidgel RA, Du X (2006). A phosphoinositide 3-kinase-AKT-nitric oxide-cGMP signaling pathway in stimulating platelet secretion and aggregation. J Biol Chem.

[66] Garcia A, Quinton TM, Dorsam RT, Kunapuli SP (2005). Src family kinase-mediated and Erk-mediated thromboxane A2 generation are essential for VWF/GPIb-induced fibrinogen receptor activation in human platelets. Blood.

[67] Li Z, Xi X, Gu M, Feil R, Ye RD, Eigenthaler M, Hofmann F, Du X (2003). A stimulatory role for cGMP-dependent protein kinase in platelet activation. Cell.

[68] Marshall SJ, Senis YA, Auger JM, Feil R, Hofmann F, Salmon G, Peterson JT, Burslem F, Watson SP (2004). GPIb-dependent platelet activation is dependent on Src kinases but not MAP kinase or cGMP-dependent kinase. Blood.

[69] Begonja AJ, Geige J, Rukoyatkina N, Rauchfuss S, Gambaryan S, Walter U (2007). Thrombin stimulation of p38 MAP kinase in human platelets is mediated by ADP and thromboxane A2 and inhibited by cGMP/cGMP-dependent protein kinase. Blood.

[70] Herve D, Philippi A, Belbouab R, Zerah M, Chabrier S, Collardeau-Frachon S, Bergametti F, Essongue A, Berrou E, Krivosic V, Sainte-Rose C, Houdart E, Adam F, Billiemaz K, Lebret M, Roman S, Passemard S, Boulday G, Delaforge A, Guey S, Dray X, Chabriat H, Brouckaert P, Bryckaert M, Tournier-Lasserve E (2014). Loss of alpha1beta1 soluble guanylate cyclase, the major nitric oxide receptor, leads to moyamoya and achalasia. Am J Hum Genet.

[71] Mazzucato M, Pradella P, Cozzi MR, De Marco L, Ruggeri ZM (2002). Sequential cytoplasmic calcium signals in a 2-stage platelet activation process induced by the glycoprotein Ibalpha mechanoreceptor. Blood.

[72] Nesbitt WS, Kulkarni S, Giuliano S, Goncalves I, Dopheide SM, Yap CL, Harper IS, Salem HH, Jackson SP (2002). Distinct glycoprotein Ib/V/IX and integrin alphaIIbbeta3-dependent calcium signals cooperatively regulate platelet adhesion under flow. J Biol Chem.

[73] Liu J, Pestina TI, Berndt MC, Jackson CW, Gartner TK (2005). Botrocetin/VWF-induced signaling through GPIb-IX-V produces TxA2 in an alphaIIbbeta3- and aggregation-independent manner. Blood.

[74] Estevez B, Stojanovic-Terpo A, Delaney MK, O’Brien KA, Berndt MC, Ruan C, Du X (2013). LIM kinase-1 selectively promotes glycoprotein Ib-IX-mediated TXA2 synthesis, platelet activation, and thrombosis. Blood.

[75] Mazzucato M, Cozzi MR, Pradella P, Ruggeri ZM, De Marco L (2004). Distinct roles of ADP receptors in von Willebrand factor-mediated platelet signaling and activation under high flow. Blood.

[76] Sullam PM, Hyun WC, Szollosi J, Dong J, Foss WM, Lopez JA (1998). Physical proximity and functional interplay of the glycoprotein Ib-IX-V complex and the Fc receptor FcgammaRIIA on the platelet plasma membrane. J Biol Chem.

[77] Wu Y, Suzuki-Inoue K, Satoh K, Asazuma N, Yatomi Y, Berndt MC, Ozaki Y (2001). Role of Fc receptor gamma-chain in platelet glycoprotein Ib-mediated signaling. Blood.

[78] Kasirer-Friede A, Cozzi MR, Mazzucato M, De Marco L, Ruggeri ZM, Shattil SJ (2004). Signaling through GP Ib-IX-V activates alpha IIb beta 3 independently of other receptors. Blood.

[79] Maxwell MJ, Dopheide SM, Turner SJ, Jackson SP (2006). Shear induces a unique series of morphological changes in translocating platelets: effects of morphology on translocation dynamics. Arterioscler Thromb Vasc Biol.

[80] Yuan Y, Kulkarni S, Ulsemer P, Cranmer SL, Yap CL, Nesbitt WS, Harper I, Mistry N, Dopheide SM, Hughan SC, Williamson D, de la Salle C, Salem HH, Lanza F, Jackson SP (1999). The von Willebrand factor-glycoprotein Ib/V/IX interaction induces actin polymerization and cytoskeletal reorganization in rolling platelets and glycoprotein Ib/V/IX-transfected cells. J Biol Chem.

[81] Ruggeri ZM, Orje JN, Habermann R, Federici AB, Reininger AJ (2006). Activation-independent platelet adhesion and aggregation under elevated shear stress. Blood.

[82] Ni H, Denis CV, Subbarao S, Degen JL, Sato TN, Hynes RO, Wagner DD (2000). Persistence of platelet thrombus formation in arterioles of mice lacking both von Willebrand factor and fibrinogen. J Clin Invest.

[83] Dubois C, Panicot-Dubois L, Gainor JF, Furie BC, Furie B (2007). Thrombin-initiated platelet activation in vivo is vWF independent during thrombus formation in a laser injury model. J Clin Invest.

[84] Bergmeier W, Piffath CL, Goerge T, Cifuni SM, Ruggeri ZM, Ware J, Wagner DD (2006). The role of platelet adhesion receptor GPIbalpha far exceeds that of its main ligand, von Willebrand factor, in arterial thrombosis. Proc Natl Acad Sci USA.

[85] Jurk K, Clemetson KJ, de Groot PG, Brodde MF, Steiner M, Savion N, Varon D, Sixma JJ, Van Aken H, Kehrel BE (2003). Thrombospondin-1 mediates platelet adhesion at high shear via glycoprotein Ib (GPIb): an alternative/backup mechanism to von Willebrand factor. FASEB J.

[86] Hechler B, Nonne C, Eckly A, Magnenat S, Rinckel JY, Denis CV, Freund M, Cazenave JP, Lanza F, Gachet C (2010). Arterial thrombosis: relevance of a model with two levels of severity assessed by histologic, ultrastructural and functional characterization. J Thromb Haemost.

[87] De Meyer SF, Schwarz T, Deckmyn H, Denis CV, Nieswandt B, Stoll G, Vanhoorelbeke K, Kleinschnitz C (2010). Binding of von Willebrand factor to collagen and glycoprotein Ibalpha, but not to glycoprotein IIb/IIIa, contributes to ischemic stroke in mice—brief report. Arterioscler Thromb Vasc Biol.

[88] De Meyer SF, Stoll G, Wagner DD, Kleinschnitz C (2012). von Willebrand factor: an emerging target in stroke therapy. Stroke.

[89] Momi S, Tantucci M, Van Roy M, Ulrichts H, Ricci G, Gresele P (2013). Reperfusion of cerebral artery thrombosis by the GPIb-VWF blockade with the Nanobody ALX-0081 reduces brain infarct size in guinea pigs. Blood.

[90] Le Behot A, Gauberti M, Martinez De Lizarrondo S, Montagne A, Lemarchand E, Repesse Y, Guillou S, Denis CV, Maubert E, Orset C, Vivien D (2014). GpIbalpha-VWF blockade restores vessel patency by dissolving platelet aggregates formed under very high shear rate in mice. Blood.

[91] Ulrichts H, Silence K, Schoolmeester A, de Jaegere P, Rossenu S, Roodt J, Priem S, Lauwereys M, Casteels P, Van Bockstaele F, Verschueren K, Stanssens P, Baumeister J, Holz JB (2011). Antithrombotic drug candidate ALX-0081 shows superior preclinical efficacy and safety compared with currently marketed antiplatelet drugs. Blood.

[92] Yamamoto H, Vreys I, Stassen JM, Yoshimoto R, Vermylen J, Hoylaerts MF (1998). Antagonism of vWF inhibits both injury induced arterial and venous thrombosis in the hamster. Thromb Haemost.

[93] Oury C, Daenens K, Hu H, Toth-Zsamboki E, Bryckaert M, Hoylaerts MF (2006). ERK2 activation in arteriolar and venular murine thrombosis: platelet receptor GPIb vs. P2X. J Thromb Haemost.

[94] Bergmeier W, Chauhan AK, Wagner DD (2008). Glycoprotein Ibalpha and von Willebrand factor in primary platelet adhesion and thrombus formation: lessons from mutant mice. Thromb Haemost.

[95] Chauhan AK, Kisucka J, Lamb CB, Bergmeier W, Wagner DD (2007). von Willebrand factor and factor VIII are independently required to form stable occlusive thrombi in injured veins. Blood.

[96] Smith NL, Rice KM, Bovill EG, Cushman M, Bis JC, McKnight B, Lumley T, Glazer NL, van Hylckama Vlieg A, Tang W, Dehghan A, Strachan DP, O’Donnell CJ, Rotter JI, Heckbert SR, Psaty BM, Rosendaal FR (2011). Genetic variation associated with plasma von Willebrand factor levels and the risk of incident venous thrombosis. Blood.

[97] Brill A, Fuchs TA, Chauhan AK, Yang JJ, De Meyer SF, Kollnberger M, Wakefield TW, Lammle B, Massberg S, Wagner DD (2011). von Willebrand factor-mediated platelet adhesion is critical for deep vein thrombosis in mouse models. Blood.

[98] Brill A, Fuchs TA, Savchenko AS, Thomas GM, Martinod K, De Meyer SF, Bhandari AA, Wagner DD (2012). Neutrophil extracellular traps promote deep vein thrombosis in mice. J Thromb Haemost.

[99] Martinod K, Wagner DD (2014). Thrombosis: tangled up in NETs. Blood.

[100] Hulstein JJ, Lenting PJ, de Laat B, Derksen RH, Fijnheer R, de Groot PG (2007). beta2-Glycoprotein I inhibits von Willebrand factor dependent platelet adhesion and aggregation. Blood.

[101] de Laat B, de Groot PG, Derksen RH, Urbanus RT, Mertens K, Rosendaal FR, Doggen CJ (2009). Association between beta2-glycoprotein I plasma levels and the risk of myocardial infarction in older men. Blood.

[102] Sheng Y, Reddel SW, Herzog H, Wang YX, Brighton T, France MP, Robertson SA, Krilis SA (2001). Impaired thrombin generation in beta 2-glycoprotein I null mice. J Biol Chem.

[103] Yasuda S, Tsutsumi A, Chiba H, Yanai H, Miyoshi Y, Takeuchi R, Horita T, Atsumi T, Ichikawa K, Matsuura E, Koike T (2000). beta(2)-glycoprotein I deficiency: prevalence, genetic background and effects on plasma lipoprotein metabolism and hemostasis. Atherosclerosis.

[104] Levy GG, Nichols WC, Lian EC, Foroud T, McClintick JN, McGee BM, Yang AY, Siemieniak DR, Stark KR, Gruppo R, Sarode R, Shurin SB, Chandrasekaran V, Stabler SP, Sabio H, Bouhassira EE, Upshaw JD, Ginsburg D, Tsai HM (2001). Mutations in a member of the ADAMTS gene family cause thrombotic thrombocytopenic purpura. Nature.

[105] Dong JF, Moake JL, Bernardo A, Fujikawa K, Ball C, Nolasco L, Lopez JA, Cruz MA (2003). ADAMTS-13 metalloprotease interacts with the endothelial cell-derived ultra-large von Willebrand factor. J Biol Chem.

[106] Nishio K, Anderson PJ, Zheng XL, Sadler JE (2004). Binding of platelet glycoprotein Ibalpha to von Willebrand factor domain A1 stimulates the cleavage of the adjacent domain A2 by ADAMTS13. Proc Natl Acad Sci USA.

[107] Shim K, Anderson PJ, Tuley EA, Wiswall E, Sadler JE (2008). Platelet-VWF complexes are preferred substrates of ADAMTS13 under fluid shear stress. Blood.

[108] Bonnefoy A, Daenens K, Feys HB, De Vos R, Vandervoort P, Vermylen J, Lawler J, Hoylaerts MF (2006). Thrombospondin-1 controls vascular platelet recruitment and thrombus adherence in mice by protecting (sub)endothelial VWF from cleavage by ADAMTS13. Blood.

[109] Chauhan AK, Motto DG, Lamb CB, Bergmeier W, Dockal M, Plaimauer B, Scheiflinger F, Ginsburg D, Wagner DD (2006). Systemic antithrombotic effects of ADAMTS13. J Exp Med.

[110] Dong JF (2007). Structural and functional correlation of ADAMTS13. Curr Opin Hematol.

[111] Matsukawa M, Kaikita K, Soejima K, Fuchigami S, Nakamura Y, Honda T, Tsujita K, Nagayoshi Y, Kojima S, Shimomura H, Sugiyama S, Fujimoto K, Yoshimura M, Nakagaki T, Ogawa H (2007). Serial changes in von Willebrand factor-cleaving protease (ADAMTS13) and prognosis after acute myocardial infarction. Am J Cardiol.

[112] Zhao BQ, Chauhan AK, Canault M, Patten IS, Yang JJ, Dockal M, Scheiflinger F, Wagner DD (2009). von Willebrand factor-cleaving protease ADAMTS13 reduces ischemic brain injury in experimental stroke. Blood.

[113] Morioka Y, Casari C, Wohner N, Cho S, Kurata S, Kitano A, Christophe OD, Lenting PJ, Li R, Denis CV, Prevost N (2014). Expression of a structurally constrained von Willebrand factor variant triggers acute thrombotic thrombocytopenic purpura in mice. Blood.

[114] James PD, Notley C, Hegadorn C, Leggo J, Tuttle A, Tinli S, Brown C, Andrews C, Labelle A, Chirinian Y, O’Brien L, Othman M, Rivard G, Rapson D, Hough C, Lillicrap D (2007). The mutational spectrum of type 1 von Willebrand disease: results from a Canadian cohort study. Blood.

[115] Goodeve A (2007). Genetics of type 1 von Willebrand disease. Curr Opin Hematol.

[116] Cumming A, Grundy P, Keeney S, Lester W, Enayat S, Guilliatt A, Bowen D, Pasi J, Keeling D, Hill F, Bolton-Maggs PH, Hay C, Collins P, Organisation UKHCD (2006). An investigation of the von Willebrand factor genotype in UK patients diagnosed to have type 1 von Willebrand disease. Thromb Haemost.

[117] Bellissimo DB, Christopherson PA, Flood VH, Gill JC, Friedman KD, Haberichter SL, Shapiro AD, Abshire TC, Leissinger C, Hoots WK, Lusher JM, Ragni MV, Montgomery RR (2012). VWF mutations and new sequence variations identified in healthy controls are more frequent in the African-American population. Blood.

[118] Jacobi PM, Gill JC, Flood VH, Jakab DA, Friedman KD, Haberichter SL (2012). Intersection of mechanisms of type 2A VWD through defects in VWF multimerization, secretion, ADAMTS-13 susceptibility, and regulated storage. Blood.

[119] O’Brien LA, Sutherland JJ, Weaver DF, Lillicrap D (2005). Theoretical structural explanation for Group I and Group II, type 2A von Willebrand disease mutations. J Thromb Haemost.

[120] Sadler JE (1991). von Willebrand factor. J Biol Chem.

[121] Sugimoto M, Matsui H, Mizuno T, Tsuji S, Miyata S, Matsumoto M, Matsuda M, Fujimura Y, Yoshioka A (2003). Mural thrombus generation in type 2A and 2B von Willebrand disease under flow conditions. Blood.

[122] Hillery CA, Mancuso DJ, Evan Sadler J, Ponder JW, Jozwiak MA, Christopherson PA, Cox Gill J, Scott Paul, Montgomery RR (1998). Type 2 M von Willebrand disease: F606I and I662F mutations in the glycoprotein Ib binding domain selectively impair ristocetin- but not botrocetin-mediated binding of von Willebrand factor to platelets. Blood.

[123] Sadler JE, Budde U, Eikenboom JC, Favaloro EJ, Hill FG, Holmberg L, Ingerslev J, Lee CA, Lillicrap D, Mannucci PM, Mazurier C, Meyer D, Nichols WL, Nishino M, Peake IR, Rodeghiero F, Schneppenheim R, Ruggeri ZM, Srivastava A, Montgomery RR, Federici AB, Working Party on von Willebrand Disease, C (2006). Update on the pathophysiology and classification of von Willebrand disease: a report of the Subcommittee on von Willebrand Factor. J Thromb Haemost.

[124] Gralnick HR, Williams S, McKeown L, Kramer W, Krutzsch H, Gorecki M, Pinet A, Garfinkel LI (1992). A monomeric von Willebrand factor fragment, Leu-504–Lys-728, inhibits von Willebrand factor interaction with glycoprotein Ib-IX [corrected]. Proc Natl Acad Sci USA.

[125] Ribba AS, Loisel I, Lavergne JM, Juhan-Vague I, Obert B, Cherel G, Meyer D, Girma JP (2001). Ser968Thr mutation within the A3 domain of von Willebrand factor (VWF) in two related patients leads to a defective binding of VWF to collagen. Thromb Haemost.

[126] Flood VH, Lederman CA, Wren JS, Christopherson PA, Friedman KD, Hoffmann RG, Montgomery RR (2010). Absent collagen binding in a VWF A3 domain mutant: utility of the VWF:cB in diagnosis of VWD. J Thromb Haemost.

[127] Pareti FI, Niiya K, McPherson JM, Ruggeri ZM (1987). Isolation and characterization of two domains of human von Willebrand factor that interact with fibrillar collagen types I and III. J Biol Chem.

[128] Bonnefoy A, Romijn RA, Vandervoort PA, Vermylen J, Hoylaerts MF (2006). von Willebrand factor A1 domain can adequately substitute for A3 domain in recruitment of flowing platelets to collagen. J Thromb Haemost.

[129] Denis C, Baruch D, Kielty CM, Ajzenberg N, Christophe O, Meyer D (1993). Localization of von Willebrand factor binding domains to endothelial extracellular matrix and to type VI collagen. Arterioscler Thromb.

[130] Castaman G, Federici AB, Tosetto A, La Marca S, Stufano F, Mannucci PM, Rodeghiero F (2012). Different bleeding risk in type 2A and 2 M von Willebrand disease: a 2-year prospective study in 107 patients. J Thromb Haemost.

[131] Ware J, Dent JA, Azuma H, Sugimoto M, Kyrle PA, Yoshioka A, Ruggeri ZM (1991). Identification of a point mutation in type IIB von Willebrand disease illustrating the regulation of von Willebrand factor affinity for the platelet membrane glycoprotein Ib-IX receptor. Proc Natl Acad Sci USA.

[132] Nurden P, Chretien F, Poujol C, Winckler J, Borel-Derlon A, Nurden A (2000). Platelet ultrastructural abnormalities in three patients with type 2B von Willebrand disease. Br J Haematol.

[133] Loffredo G, Baronciani L, Noris P, Menna F, Federici AB, Balduini CL (2006). von Willebrand disease type 2B must be always considered in the differential diagnosis of genetic thrombocytopenias with giant platelets. Platelets.

[134] Lopez-Fernandez MF, Lopez-Berges C, Martin-Bernal JA, Sanchez R, Villaron LG, Diez-Jarilla J, Batlle J (1988). Type IIB von Willebrand’s disease associated with a complex thrombocytopenic thrombocytopathy. Am J Hematol.

[135] Moll S, Lazarowski AR, White GC (1998). Giant platelet disorder in a patient with type 2B von Willebrand’s disease. Am J Hematol.

[136] Federici AB, Mannucci PM, Castaman G, Baronciani L, Bucciarelli P, Canciani MT, Pecci A, Lenting PJ, De Groot PG (2009). Clinical and molecular predictors of thrombocytopenia and risk of bleeding in patients with von Willebrand disease type 2B: a cohort study of 67 patients. Blood.

[137] Nurden P, Gobbi G, Nurden A, Enouf J, Youlyouz-Marfak I, Carubbi C, La Marca S, Punzo M, Baronciani L, De Marco L, Vitale M, Federici AB (2010). Abnormal VWF modifies megakaryocytopoiesis: studies of platelets and megakaryocyte cultures from patients with von Willebrand disease type 2B. Blood.

[138] Rayes J, Hollestelle MJ, Legendre P, Marx I, de Groot PG, Christophe OD, Lenting PJ, Denis CV (2010). Mutation and ADAMTS13-dependent modulation of disease severity in a mouse model for von Willebrand disease type 2B. Blood.

[139] Casari C, Du V, Wu YP, Kauskot A, de Groot PG, Christophe OD, Denis CV, de Laat B, Lenting PJ (2013). Accelerated uptake of VWF/platelet complexes in macrophages contributes to VWD type 2B-associated thrombocytopenia. Blood.

[140] Li S, Wang Z, Liao Y, Zhang W, Shi Q, Yan R, Ruan C, Dai K (2010). The glycoprotein Ibalpha-von Willebrand factor interaction induces platelet apoptosis. J Thromb Haemost.

[141] van der Wal DE, Du VX, Lo KS, Rasmussen JT, Verhoef S, Akkerman JW (2010). Platelet apoptosis by cold-induced glycoprotein Ibalpha clustering. J Thromb Haemost.

[142] Morowski M, Vogtle T, Kraft P, Kleinschnitz C, Stoll G, Nieswandt B (2013). Only severe thrombocytopenia results in bleeding and defective thrombus formation in mice. Blood.

[143] Milton JG, Frojmovic MM, Tang SS, White JG (1984). Spontaneous platelet aggregation in a hereditary giant platelet syndrome (MPS). Am J Pathol.

[144] Jackson SC, Sinclair GD, Cloutier S, Duan Z, Rand ML, Poon MC (2009). The Montreal platelet syndrome kindred has type 2B von Willebrand disease with the VWF V1316M mutation. Blood.

[145] Casari C, Berrou E, Lebret M, Adam F, Kauskot A, Bobe R, Desconclois C, Fressinaud E, Christophe OD, Lenting PJ, Rosa JP, Denis CV, Bryckaert M (2013). von Willebrand factor mutation promotes thrombocytopathy by inhibiting integrin alphaIIbbeta3. J Clin Invest..

[146] Stefanini L, Roden RC, Bergmeier W (2009). CalDAG-GEFI is at the nexus of calcium-dependent platelet activation. Blood.

[147] Zwartkruis FJ, Bos JL (1999). Ras and Rap1: two highly related small GTPases with distinct function. Exp Cell Res.

[148] Guidetti GF, Manganaro D, Consonni A, Canobbio I, Balduini C, Torti M (2013). Phosphorylation of the guanine-nucleotide-exchange factor CalDAG-GEFI by protein kinase A regulates Ca(2+)-dependent activation of platelet Rap1b GTPase. Biochem J.

[149] Schultess J, Danielewski O, Smolenski AP (2005). Rap1GAP2 is a new GTPase-activating protein of Rap1 expressed in human platelets. Blood.

[150] Hoffmeister M, Riha P, Neumuller O, Danielewski O, Schultess J, Smolenski AP (2008). Cyclic nucleotide-dependent protein kinases inhibit binding of 14-3-3 to the GTPase-activating protein Rap1GAP2 in platelets. J Biol Chem.

[151] Ware J (2013). Thrombocytopathy and type 2B von Willebrand disease. J Clin Invest.

[152] Miller JL, Castella A (1982). Platelet-type von Willebrand’s disease: characterization of a new bleeding disorder. Blood.

[153] Miller JL (1996). Platelet-type von Willebrand disease. Thromb Haemost.

[154] Othman M, Lopez JA, Ware J (2011). Platelet-type von Willebrand disease update: the disease, the molecule and the animal model. Expert Rev Hematol.

[155] Matsubara Y, Murata M, Sugita K, Ikeda Y (2003). Identification of a novel point mutation in platelet glycoprotein Ibalpha, Gly to Ser at residue 233, in a Japanese family with platelet-type von Willebrand disease. J Thromb Haemost.

[156] Russell SD, Roth GJ (1993). Pseudo-von Willebrand disease: a mutation in the platelet glycoprotein Ib alpha gene associated with a hyperactive surface receptor. Blood.

[157] Takahashi H, Murata M, Moriki T, Anbo H, Furukawa T, Nikkuni K, Shibata A, Handa M, Kawai Y, Watanabe K (1995). Substitution of Val for Met at residue 239 of platelet glycoprotein Ib alpha in Japanese patients with platelet-type von Willebrand disease. Blood.

[158] Suva LJ, Hartman E, Dilley JD, Russell S, Akel NS, Skinner RA, Hogue WR, Budde U, Varughese KI, Ware J, Kanaji (2008). Platelet dysfunction and a high bone mass phenotype in a murine model of platelet-type von Willebrand disease. Am J Pathol.

[159] Guerrero JA, Kyei M, Russell S, Liu J, Gartner TK, Storrie B, Ware J (2009). Visualizing the von Willebrand factor/glycoprotein Ib-IX axis with a platelet-type von Willebrand disease mutation. Blood.

[160] Jackson SP, Schoenwaelder SM, Goncalves I, Nesbitt WS, Yap CL, Wright CE, Kenche V, Anderson KE, Dopheide SM, Yuan Y, Sturgeon SA, Prabaharan H, Thompson PE, Smith GD, Shepherd PR, Daniele N, Kulkarni S, Abbott B, Saylik D, Jones C, Lu L, Giuliano S, Hughan SC, Angus JA, Robertson AD, Salem HH (2005). PI 3-kinase p110beta: a new target for antithrombotic therapy. Nat Med.

